# Iron and Manganese Azooxime Complexes as Anti‐Microbial Agents Against Antibiotics Resistant Wild Bacteria From Hospital Drainage

**DOI:** 10.1111/jcmm.70826

**Published:** 2025-09-05

**Authors:** Aratrika Samajdar, Supriyo Halder, Sukanya Chatterjee, Debjeet Chakraborty, Arup Kumar Mitra, Anindita Banerjee, Kausikisankar Pramanik, Sanjib Ganguly, Ajoy Kumer, Bikram Dhara

**Affiliations:** ^1^ Department of Microbiology St. Xavier's College (Autonomous) Kolkata India; ^2^ Department of Chemistry Jadavpur University Kolkata India; ^3^ Department of Chemistry St. Xavier's College (Autonomous) Kolkata India; ^4^ Department of Chemistry IUBAT‐International University of Business Agriculture & Technology Dhaka Bangladesh; ^5^ Centre for Global Health Research Saveetha Medical College and Hospital, Saveetha Institute of Medical and Technical Sciences Chennai India

**Keywords:** azo‐oxime, catalase, coagulase, lipid peroxidation, multi drug resistant

## Abstract

Antibiotic resistance is the never‐ending war among medical researchers and microbial life forms. The extensive evolving potential of the microorganisms, in combination with improper usage, storage and disposal of the marketed antibiotics generated from natural or artificial sources, always calls for the need for novel antimicrobial agents with different modes of action. In this project, azo‐oxime complexes of iron and manganese (seven in total) have been applied to wild multidrug‐resistant pathogenic bacterial strains (isolated from sewage water of hospital). All complexes were inhibitory to bacterial strains present in the sewage water sample, which have been authenticated by a significant reduction in colony count upon their application to the microbial population of the water sample. Four of the most abundant colonies were isolated for further investigation about the bacterial characteristics, as well as to comprehend the molecular mechanism of action of these complexes to inhibit bacterial growth. Biochemical experiments in the form of the Catalase test, Coagulase test and lipase assay point towards the pathogenicity of bacterial strains. The strains were treated with various broad‐spectrum antibiotics, namely, Penicillin G, Oxacillin, Cephalothin, Clindamycin, Erythromycin, Amoxyclav, Cefotaxime, Levofloxacin, Aztreonam, Imipenem, Amikacin, Ceftazidime, and found to be resistant against many of them, viz., Clindamycin, Ceftazidime, Erythromycin, Amoxyclav, and some others, thereby signifying that the molecular mechanism of action of the aforesaid complexes is multidimensional. These complexes were producing ROS in sufficient amounts that can cause lipid peroxidation, and subsequent damage to the bacterial cell membrane and translation machinery was found to be inhibited by RNA. Bacterial genomic DNA was also affected by the chelates, and this has been authenticated by the decreased genomic DNA concentration and presence of DNA debris on agarose gel electrophoresis of the DNA of bacterial cultures treated with the complexes.

## 1 Introduction

Antibiotics have proven to be of vital importance among medical specialists in dealing with life‐threatening bacterial infections for the past seven to eight decades [1, 2]. These drugs have been used in an unrestrained/inappropriate manner for quite some time, and their indelicate disposal practices by human civilisation have led to the spreading of multidrug‐tolerant bacteria in soil as well as in waterbodies [1, 3]. Currently, anti‐microbial resistance as well as multi‐drug‐resistant (MDR) bacteria have emerged as a global threat in medical science, and in 2019, the World Health Organization (WHO) reported a death toll of 700,000 people all over the world [1, 2, 3, 4, 5, 6] owing to antimicrobial resistance. The anti‐tuberculosis drug resistance monitoring outcomes have recently reported that nearly 3.5% of the tuberculosis cases are suspected to be due to MDR [1]. During the COVID‐19 upsurge, the concern of anti‐microbial resistance was escalated due to extensive consumption of a wide variety of antibiotics [7]. In another study (in Greece), it was found that Coagulase‐negative *Staphylococci* led to the majority of the infections of intravenous catheters correlated with bacteraemia. They have been found to display 72% insusceptibility for ampicillin, oxacillin, ceftazidime, ceftriaxone, cefaclor, amoxicillin and imipenem [8]. This serious societal concern therefore demands a dedicated and untiring investigation on exploration for new drugs that can provide some relief from wild MDR bacteria.

**SCHEME 1:**
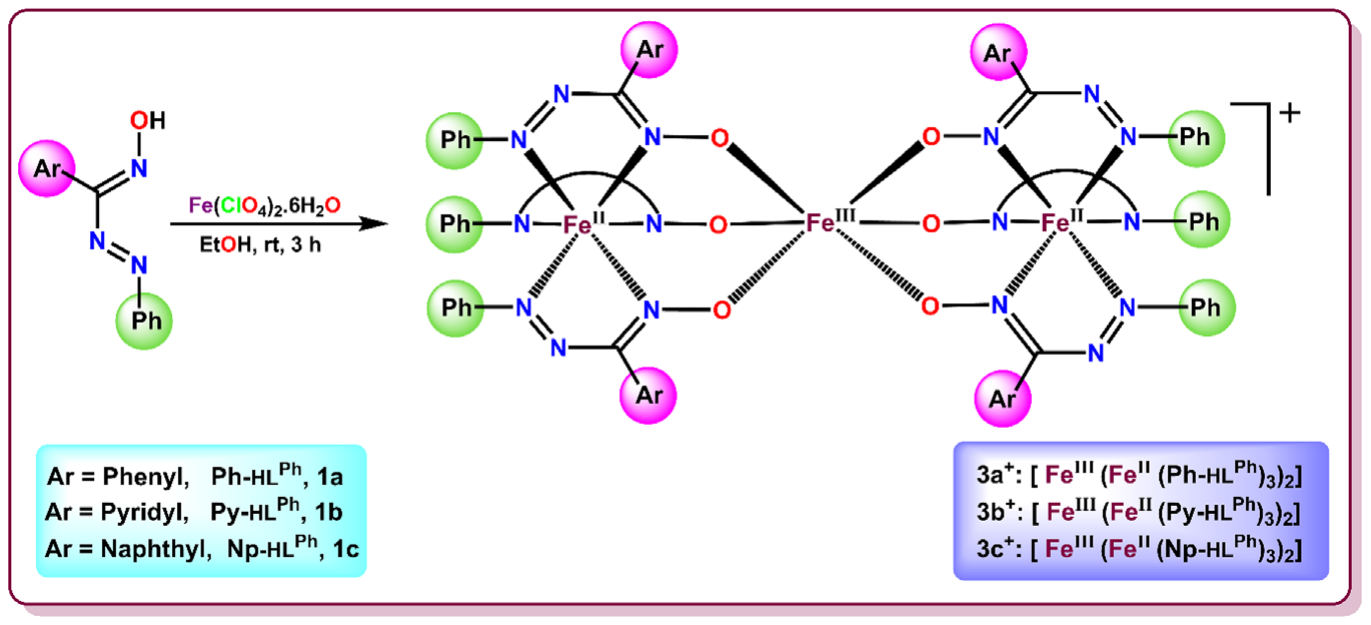
Synthetic strategy for tri‐nuclear Fe complexes with arylazooxime.

**SCHEME 2:**
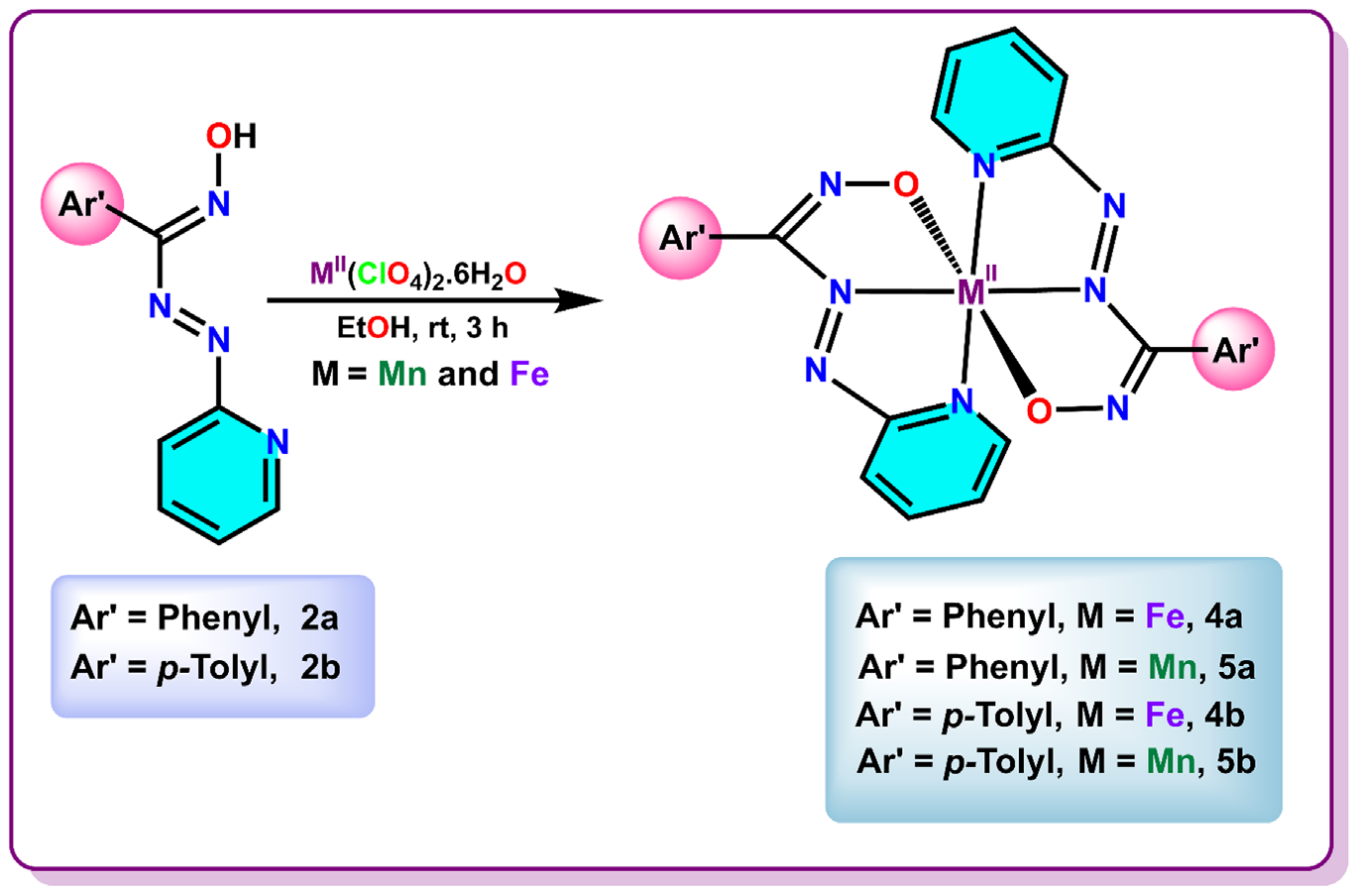
Synthetic strategy for bis complexes of Fe and Mn with pyridylazooxime.

To throw some light on this challenging problem, we have focussed on combating multidrug‐resistant and disease‐causing bacteria by using seven coordination complexes of iron and manganese with azo‐oxime type ligands (Schemes 1 and 2), since they may be quite promising in inhibiting the growth of long‐range microbial strains [9, 10]. Both the metal ions act as micronutrients in the human body and are hence less detrimental when used as drug components. They are also involved in several beneficial functions in the human body, like glucose homeostasis, enzyme functionality and hence their deficiency can lead to malnutrition as well as other distressful conditions [11, 12] Additionally, both have shown efficacy in inhibiting pathogenic Gram‐positive and Gram‐negative bacteria [12, 13]. All seven transition metal complexes have efficaciously inhibited bacterial growth, and this has been observed in their reduced colony count (in vitro) upon administration. The bacterial strains present in the water sample (sewage water near a hospital compound) revealed that the most abundant bacterial colonies (*Escherichia coli, Stenotrophomonas maltophilia, Micrococcus luteus, Bacillus anthracis*) were not only pathogenic, but also resistant to multiple broad‐spectrum antibiotics like vancomycin, amoxiclav, etc.

Both the iron and manganese complexes have been found to exhibit superior activity, and the former has even more efficient anti‐microbial activity. Inhibition of bacterial growth occurs by effecting multiple domains in bacterial strains. Both types of complexes have led to significant oxidative stress in the bacterial cellular environment by producing large amounts of ROS and subsequently causing lipid peroxidation, membrane damage, and producing DNA debris via DNA damage. The translation machinery was also found to be affected through inactivation of RNAs. Actual environmental sources of MDRs, like waste water, hospital drainage, soils of dumping grounds, and industrial wastes are still less explored [14] and the results are that we have focused on the annihilation of wild multidrug‐resistant bacterial populations from natural water samples collected from hospital sewage, and we have successfully employed the seven chelate complexes on wild, uncharacterised, pathogenic bacterial populations.

## 2 Methodology

The reagents used in the experiments are mostly from Himedia. All the necessary biosafety guidelines have been followed while sample collection, handling the bacterial cultures, and performing the tests.

### 2.1 Sample Collection

The water samples were collected from the sewer in Bidhannagar Govt. hospital, (Kolkata, West Bengal, India) campus. Sterilised containers were used to collect, carry and store the water samples. 500 mL of water sample was collected. The surrounding atmospheric temperature was ~30°C. To maintain the optimal condition, the water samples were applied to the growth media as soon as possible. Disposable surgical gloves were used all the time to avoid any hygienic problem.

### 2.2 Observation of the Wholistic Effect of the Metal Complexes on Wild Bacterial Strains in Natural (Sewage Water) Sample and Isolation of Affected Bacterial Strains

Sewage water samples were diluted (10^−3^ dilution) and used in spread plate on nutrient agar media containing 70 ppm of metal complexes. The control set was also prepared without adding any complexes to the media. Colonies were counted after overnight incubation at 37°C. Four bacterial colonies that were observed to be reduced in number on the plates containing metal complexes were isolated for further investigation.

### 2.3 Identification of Wild Bacterial Strains

The bacterial cultures were identified through 16S rRNA analysis. Genomic DNA was extracted from the bacterial pure cultures and the 16S rRNA gene was isolated and purified by PCR amplification. The forward and reverse DNA sequencing was done by using primers and BDT v3.1 cycle sequencing kit on ABI 3730xl Genetic Analyser. The 16S rRNA sequences were used to carry out BLAST in the NCBI gene bank database and the phylogenetic trees were constructed using MEGA 10 software.

### 2.4 Catalase Test

Standard tube protocol [15] of applying 3% H_2_O_2_ on bacterial cultures was followed to identify catalase‐producing bacterial strains. Catalase‐negative *Streptococcus mutans* culture was used as control.

### 2.5 Coagulase Test

In this project, goat blood was used to test the presence of the afore said enzyme in isolated bacterial strains. As the standard protocol goes, [16] blood plasma was separated and bacterial culture was added to observe the clear solution become opaque due to coagulation after 4 to 6 h of incubation at 37°C.

### 2.6 Lipase Assay

For lipase assay, bacterial cultures were applied on tributyrin agar plates. The appearance of a clear zone around bacterial colonies indicated the presence of lipase enzyme [17].

### 2.7 Capsule Staining

Capsule staining of *B. anthracis* by nigrosine and crystal violet was done using standard protocol [18]. The halo zone surrounding pink (stained by crystal violet) cells on a dark blue background (stained by nigrosine) indicated the presence of a capsule.

### 2.8 Antibiotic Susceptibility Test

Antibiotic susceptibility of the four bacterial cultures was determined through the antibiotic disc diffusion method [19]. Hexa multidiscs (HX001 and HX010) were used on the bacterial cultures.

### 2.9 Determination of IC50 Values of the Metal Complexes for Different Bacterial Cultures

Reduction of bacterial growth and respective IC50 values of the metal complexes for all four bacterial strains was measured by CFU counting. Bacterial cultures were applied on the nutrient agar plates containing different strengths (10, 30, 50, 70, 100 ppm) of the metal complexes, and colonies were counted after overnight incubation at 37°C. The difference in colony counts was recorded with respect to the control sets without any metal complexes. The metal complexes were dissolved in DCM (dichloromethane) which had a little negative effect on bacterial growth; hence, the control sets were treated with 100 ppm of DCM to nullify interference from the solvent. The strength of the complexes that inhibited 50% of bacterial growth is noted as IC50 values of a particular complex for a particular bacterial strain. The percent reduction of growth was calculated as = [(CFU of solvent control − CFU of test set)/CFU of solvent control] × 100. The antibacterial efficacy of the metal complexes was also compared to the effect of the standard broad‐spectrum antibiotic (Tetracycline).

### 2.10 Effect of Ligands and Metallic Salts on the Bacterial Cultures

The effect of the ligands and the metallic salts (Fe(II) perchlorate and Mn(II) perchlorate) used to synthesise the complexes was measured on each bacterial strain in similar ways as described above.

### 2.11 Screening for the Bactericidal Effect of the Metal Complexes

The bactericidal effect of the metal complexes on the bacterial cultures was measured by CFU count method. Bacterial broth culture incubated with different strengths of metal complexes was transferred to fresh agar media and incubated overnight under optimal growth conditions. When the bacterial strains could not recover their usual growth on fresh media, it indicates the bactericidal effect of the complexes.

### 2.12 Molecular Weight and Molecular Mechanism of Action of the Metallic Complexes

Seven metallic complexes in this project can use different and more than one way to inhibit these multi drug resistant, pathogenic bacterial cultures. To identify those ways, different molecular biological tests have been applied.

### 2.13 Molecular Docking

The molecular docking experiments were performed with some of the metal complexes (complex 4a, complex 5a, complex 5b) having defined 3D structures targeting two crucial macromolecules i.e., 30S ribosomal subunit and DNA gyrase of *E. coli* . The X‐ray crystallographic structures of 30S ribosomal subunit (PDB ID: 7oe1) and DNA gyrase (PDB IB: 6RKS), of *E. coli* were taken. The structures were further processed by eliminating water molecules followed by adding polar hydrogens and Kollman charges in MGL AutoDock Tools [20] thereby converting the receptor macromolecule into PDBQT format. The configuration file for both the receptor macromolecules was specified with the dimensions of a rectangular grid box of 120 Å × 120 Å × 120 Å assigned to them encompassing all the potential active sites.

The crystallographic structures of the complexes were processed in MGL AutoDock Tools [20] by adding polar hydrogens and converting them into PDBQT files. A configuration file was generated containing the ligand and the receptor information as well as the size and location of the rectangular grid box in 3‐dimensional space.

The docking was performed using AutoDock Vina [20] built in MGL AutoDock Tools along with default parameters. The global search exhaustiveness was set to 16, and the maximum number of binding modes was set to 10 [21].

The best optimised docking pose with minimum energy and RMSD 0.0 was chosen and analysed. The docking images were then generated using Chimera version 1.17.3.

### 2.14 Lipid Peroxidation Assay or TBARS Assay

TBARS or thiobarbituric acid reactive species assay [22] has been done to detect peroxidation of lipids in bacterial cultures. Peroxidation of phospholipids would eventually harm the cell membrane and produce MDA (malondialdehyde). The standard assay protocols have been used to measure MDA concentration, which is directly proportional to the extent of lipid peroxidation.

### 2.15 NBT (Nitrobluetetrazolium) Assay to Measure ROS Production

0.01% NBT stock solution was prepared in PBS. Working concentration of NBT was prepared by 1:10 dilution of the same in PBS. 500 μL of NBT working solutions were added to 500 μL of bacterial cultures treated with metal complexes and incubated for 45 min at 37°C. The reaction mixtures were then centrifuged and the cell pellets were washed twice with PBS to exclude residual NBT. The concentration of formazan produced in the cells due to the reduction of NBT represents the amounts of ROS produced in the system. To quantify the formazan, 300 μL of 2M KOH dissolved in DMSO was added and the resulting blue coloured solutions were measured spectrophotometrically at 630 nm [23]. The concentrations of formazan were calculated with reference to the of NBT standard curve. To prepare the standard curve, formazan crystals were produced by adding 25 mg of NBT in 1 mL sodium ascorbate solution (150 mg sodium ascorbate in 1 mL distilled water). Upon stirring for 5 min, the resulting formazan crystals were isolated by filtration followed by drying and weighing [24].

### 2.16 Protein and DNA Leakage Assay

Protein and DNA leakage assay was done to point towards membrane damage of the bacterial cells due to lipid peroxidation by the metal complexes. In this assay, protein as well as DNA concentrations were measured in the extracellular fluid after incubating the bacterial cultures with the metal complexes. The same had been done with the extracellular fluids of the untreated cultures also. Increase in the protein and nucleic acid concentration in the extracellular fluid of the bacterial cultures upon treatment with the metal complexes would indicate membrane damage which might have caused leakage of intracellular protein and DNA into extracellular fluid.

### 2.17 Measurement of Total Protein Concentration

Cell‐free extract of bacterial broth culture upon incubation with metallic complexes was used as the source of bacterial total protein [25]. The protein concentrations were measured in a Nanodrop spectrophotometer [26].

### 2.18 In Vitro Translation Assay

In this experiment, the protein synthesis process of the bacterial strains was made to happen out of the cells, with all the required ingredients separately isolated from the cells in treatment as well as in untreated conditions. As mentioned above, cell‐free extracts of bacterial strains were used to measure total protein concentrations by Nanodrop spectrophotometer. To accomplish the translation process, bacterial cell‐free extracts (without any denaturing agent) were incubated with total RNA (isolated separately from both treated and untreated bacterial cultures), an amino acid mixture (40 μM each of 20 amino acids), 2 mM ATP, 0.2 mM GTP, and metal complexes for 1 h at 25°C. The total protein concentrations were measured again by Nanodrop spectrophotometer [27, 28]. Control sets were prepared without any metal complex. Values were compared between treated and untreated reaction sets in respect to the increment of total protein concentrations from the previously measured values.

In the next step, the experiment was repeated with a small change. In all the test sets containing metal complexes, the total RNA isolated from the complex treated bacterial cultures was replaced by the total RNA isolated from the untreated bacterial cultures. Upon 1 h incubation the total protein concentrations were measured again and the values were compared with the previously recorded total protein concentration values.

### 2.19 Genomic DNA Extraction and Agarose Gel Electrophoresis

Overnight grown log phase bacterial broth cultures were treated with the metal complexes. HipurA bacterial genomic DNA extraction kit was used to isolate bacterial genomic DNA. The DNA concentrations were measured using a Nanodrop spectrophotometer Multiscan Sky [29]. The mentioned spectrophotometer uses the natural surface tension property of the sample, instead of the conventional cuvette‐based method to determine the concentration of the biomolecule. To determine the concentration, first 1 μL buffer is placed on the lower optical surface of the machine to measure the blank, then 1 μL DNA was placed directly on the lower optical surface of the instrument and, with the help of fibre optics technology, the machine measures the concentration of the biomolecule and shows the values digitally on the machine screen. By assessing absorbance at 260/280 nm as well as at 260/230 nm, the software analyses the purity of the DNA sample and eliminates protein and RNA contamination respectively. A standard protocol for agarose gel electrophoresis has been used to detect any DNA damage.

### 2.20 Electrophoretic Mobility Shift Assay (EMSA)

Electrophoretic mobility shift assay was done to detect if the metal complexes are directly binding with the bacterial genomic DNA [30]. Genomic DNA isolated from the bacterial cultures was mixed with different metal complexes and run in agarose gel electrophoresis alongside the bacterial genomic DNA without mixing with metal complexes. Upon binding to the genomic DNA, the metal complexes would make the genomic DNA bulkier than its pure form and hence would run slower in the agarose gel, resulting in band shift.

## 3 Results

### 3.1 Wholistic Effect of the Complexes on Wild Bacterial Strains in Sewage Water Sample

All the nutrient agar plates containing metallic complexes exhibited huge reduction in colony count, compared to that of the control plate with no metallic complexes (Figure S1). Maximum reduction in CFU (colony forming unit) found in the plate containing 70 ppm of complex 3c. NA plates with Mn‐containing metal complexes (complex 4b and complex 5b) had maximum colonies. The observations indicate that all the seven metallic complexes are able to control bacterial growth in the water sample in a wholistic manner (Table S1). Fe‐containing complexes are somewhat more effective than Mn‐containing complexes.

### 3.2 Identification of Wild Bacterial Strains

The results of 16S rRNA revealed that the four wild bacterial colonies isolated from the hospital drainage were *E. coli, S. maltophilia, M. luteus* and *B. anthracis*. The GeneBank accession numbers are as follows—SUB14901566 Consensus PQ662462, https://submit.ncbi.nlm.nih.gov/subs/?search=SUB14901566; SUB14901458 Consensus PQ662461, https://submit.ncbi.nlm.nih.gov/subs/?search=SUB14901458.

### 3.3 Characterisation of Wild Bacterial Strains

Pure cultures of above‐mentioned bacterial strains have been used for further bacterial characterisation.

### 3.4 Catalase Test

All four bacterial cultures exhibited positive results in the catalase test, as they formed bubbles instantly in H_2_O_2_ solution (Figure S2), indicating that all four cultures produce the catalase enzyme, hence to some extent resistant to the oxidative stress produced by antimicrobial agents, which points towards probable pathogenicity.

### 3.5 Coagulase Test

Apart from *S. maltophilia*, all three bacterial cultures were found to produce coagulase enzyme that caused blood coagulation, and the goat blood plasma became opaque (Figure S3). The ability to coagulate mammalian blood samples makes the bacterial cultures pathogenic. On top of that, the clots produced by the bacteria act as a cloak around the microbes and shield them from the phagocytosis of the host immune system.

### 3.6 Lipase Assay

Among all four bacterial strains, only *S. maltophilia* showed clear zones around the bacterial colonies on tributyrin agar plates. The ability to produce lipase indicates probable pathogenicity of the bacterial strain (Figure S5).

### 3.7 Capsule Staining

In *B. anthracis*, a halo zone surrounding pink (stained by crystal violet) cells on a dark blue background (stained by nigrosine) indicates the presence of a capsule. As the virulent gene of *B. anthracis* is associated with the capsule‐producing gene, the presence of a capsule confirms the pathogenicity of the bacterial strain [31] (Figure S8).

### 3.8 Antibiotic Susceptibility Test

All four bacterial cultures were tested for their antibiotic susceptibility by using Hexa multi discs (HiMedia). *B. anthracis* strain isolated from hospital drainage water exhibited resistance against Clindamycin, Aztreonam. *S. maltophilia* was found to be resistant against Ceftazidime. *E. coli* was resistant against Penicillin G, Oxacillin, Cephalothin, Clindamycin, Erythromycin, Amoxyclav, Ceftazidime. *M. luteus* was resistant against Clindamycin and Amoxyclav (Figure S4).

### 3.9 Determination of IC50 Values of the Metal Complexes for Different Bacterial Cultures

**FIGURE 1:**
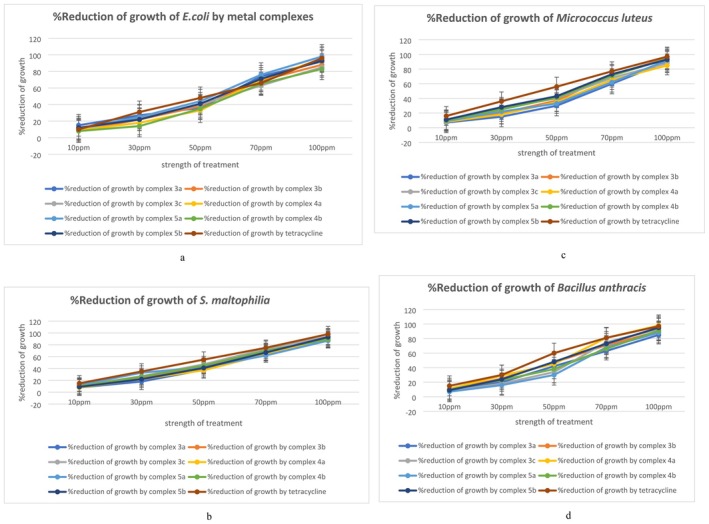
Increase in bacterial growth reduction with increasing strength of the metal complexes.

In all four bacterial strains, the growth reductions were found to be increasing with increasing strength of the metal complexes, for all seven transition metal complexes (Figure 1 and Table S2a–d). All seven metal complexes exhibited similar efficacy on the bacterial growth as shown by Tetracycline. High antibacterial efficacy of the metal complexes was also confirmed by the IC50 values of the complexes for different bacterial strains. All seven metal complexes have IC50 values under 70 ppm for all four bacterial strains (Table 1).

**TABLE 1:** IC50 values of the metal complexes for different bacterial strains.

Name of the complexes	IC50 values for *Escherichia* *coli*	IC50 values for *Micrococcus* *luteus*	IC50 values for *Stenotrophomonas* *maltophilia*	IC50 values for *Bacillus* *anthracis*
Complex 3a	60 ppm	65 ppm	55 ppm	55 ppm
Complex 3b	65 ppm	60 ppm	52 ppm	51 ppm
Complex 3c	68 ppm	62 ppm	51 ppm	57 ppm
Complex 4a	60 ppm	60 ppm	55 ppm	51 ppm
Complex 5a	55 ppm	60 ppm	52 ppm	60 ppm
Complex 4b	60 ppm	52 ppm	52 ppm	62 ppm
Complex 5b	55 ppm	52 ppm	55 ppm	51 ppm

### 3.10 Effect of Ligands and Metallic Salts on the Bacterial Cultures

Among all five ligands, only ligand 1b exhibited little antibacterial effect against *M. luteus*. The antibacterial efficacy of the ligand was negligible when compared to the respective metal complex on the same bacterial strain (Figure S6). Inhibitory effects of the salts (Fe(II) perchlorate and Mn(II) perchlorate) were also far less than those of the respective complexes (Figure S7a,b).

### 3.11 Screening for the Bactericidal Effect of the Metal Complexes

**FIGURE 2:**
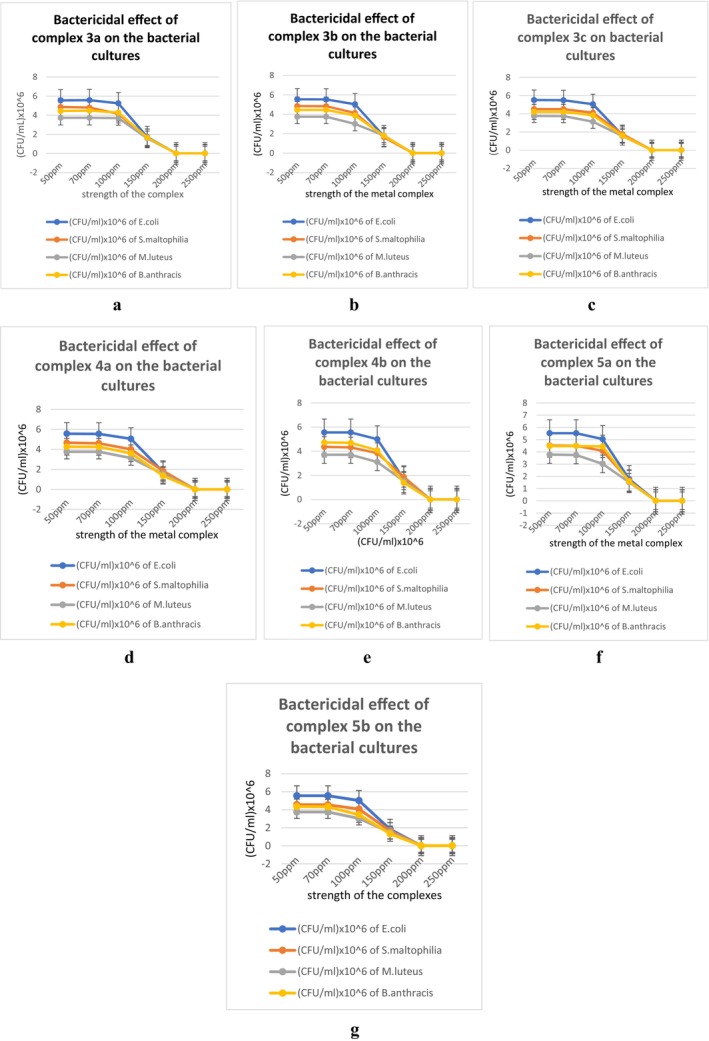
Bactericidal effect of the metal complexes when applied in higher concentration (200 ppm and above).

The seven transition metal complexes appeared bacteriostatic towards all four bacterial strains up to 100 ppm of strength, as up to this strength of the complexes the bacterial cultures exhibited normal growth as the untreated cultures when applied on fresh media after the treatment. At 150 ppm the metal complexes started to show bactericidal effect which was reflected as a huge decline in the CFU count when applied on fresh media after the treatment and at 200 ppm and above that (250 ppm), only a negligible amount of bacterial colonies was found on the fresh media, confirming complete bactericidal effect by the metal complexes (Table S3a–g and Figure 2).

### 3.12 Molecular Weight and Molecular Mechanism of Action of the Metallic Complexes

All seven azo‐oxime complexes inhibit the growth of these four antibiotic‐resistant pathogenic bacterial cultures. To determine the probable molecular mechanism of growth inhibition by the complexes, various experiments have been performed. The complexes 3a, 3b and 3c did not show crystalline structures; hence, their exact molecular weight can not be calculated, but the molecular weight of the ligands (1a, 1b, 1c) can be shown (Table 2).

**TABLE 2:** Molecular weight of ligand 1a, 1b, 1c and complex 4a, 5a, 4b, 5b.

Name of ligands/complexes	Molecular weight
Ligand 1a	1612.4390
Ligand 1b	1618.3670
Ligand 1c	1912.7990
Complex 4a	506.3070
Complex 5b	505.40
Complex 4b	534.3610
Complex 5b	533.4540

### 3.13 Molecular Docking

**FIGURE 3:**
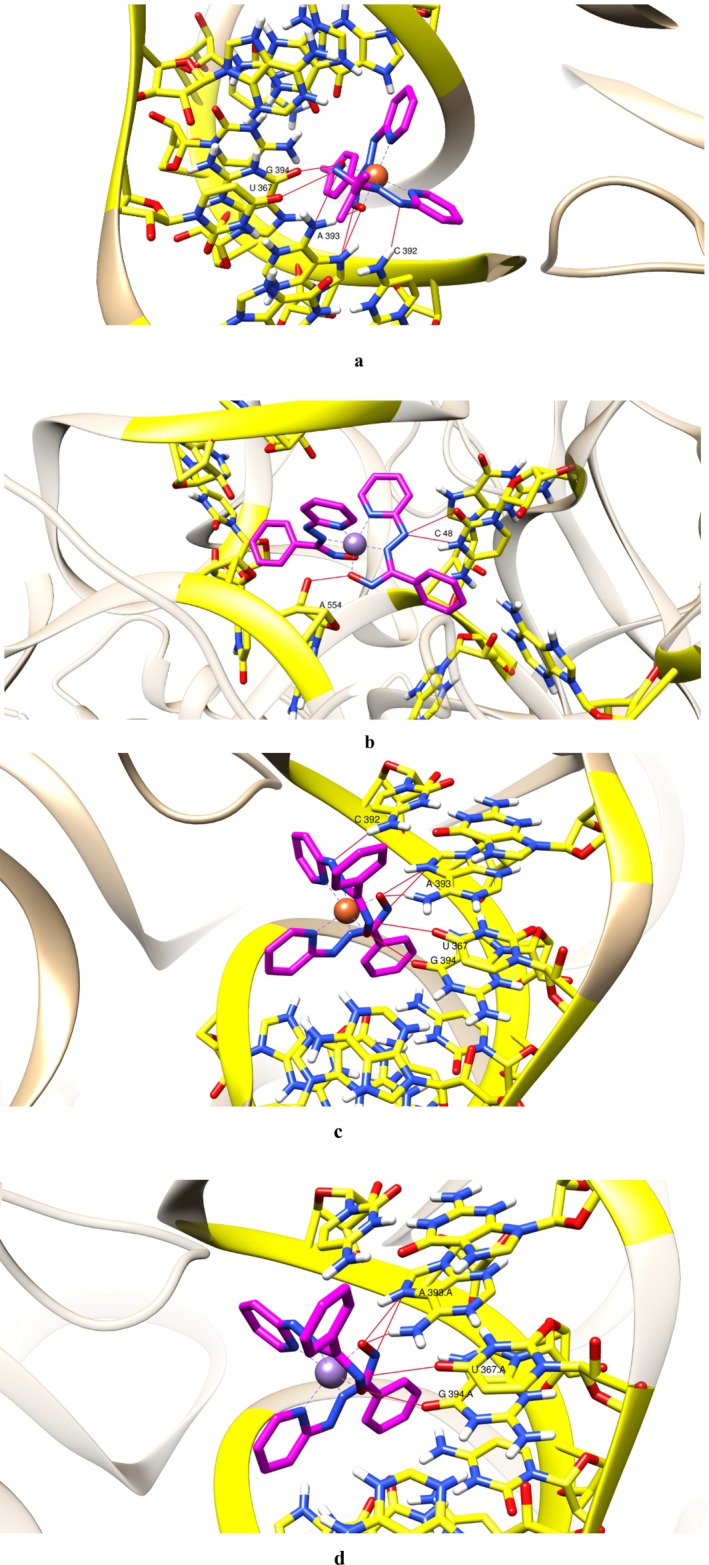
(a) Binding interactions of complex 5a (highlighted in magenta) and Chain A (16S rRNA) of 30S ribosomal subunit of *Escherichia coli* forming H bonds (shown in red) with A554 and C48. (b) Binding interactions of complex 5b (highlighted in magenta) and Chain A (16S rRNA) of 30S ribosomal subunit of *E. coli* forming H bonds (shown in red) with G394, U367, A393 and C393 (shown in yellow). (c) Binding interactions of Fe_2_HL2 complex (highlighted in magenta) and Chain A of 30S ribosomal subunit of *E. coli* forming H bonds (shown in red) with C 392, A 393, U 367 and G 394 (H bond restraint 0.4 Å and 20°). (d) Binding interactions of complex 4b (highlighted in magenta) and Chain N of 30S ribosomal subunit of *E. coli* forming H bonds (shown in red) with GLN 65 and ARG 64 (H bond restraint 0.4 Å and 120°).

The binding affinity interactions generated in the experiment suggest that the complexes have high binding affinity for ribosomal 30S subunit on 16S rRNA and moderate binding affinity for DNA gyrase (Figures 3 and S11; Table 3).

**TABLE 3:** Binding affinity interactions generated from AutoDock Vina.

Structure	Affinity (kcal/mol) 30S ribosomal subunit	Affinity (kcal/mol) DNA gyrase
Complex 5a	−12.0	−8.6
Complex 5b	−11.6	−9.4
Complex 4a	−12.5	−9.3
Complex 4b	−12.5	−9.2

### 3.14 TBARS Assay

**FIGURE 4:**
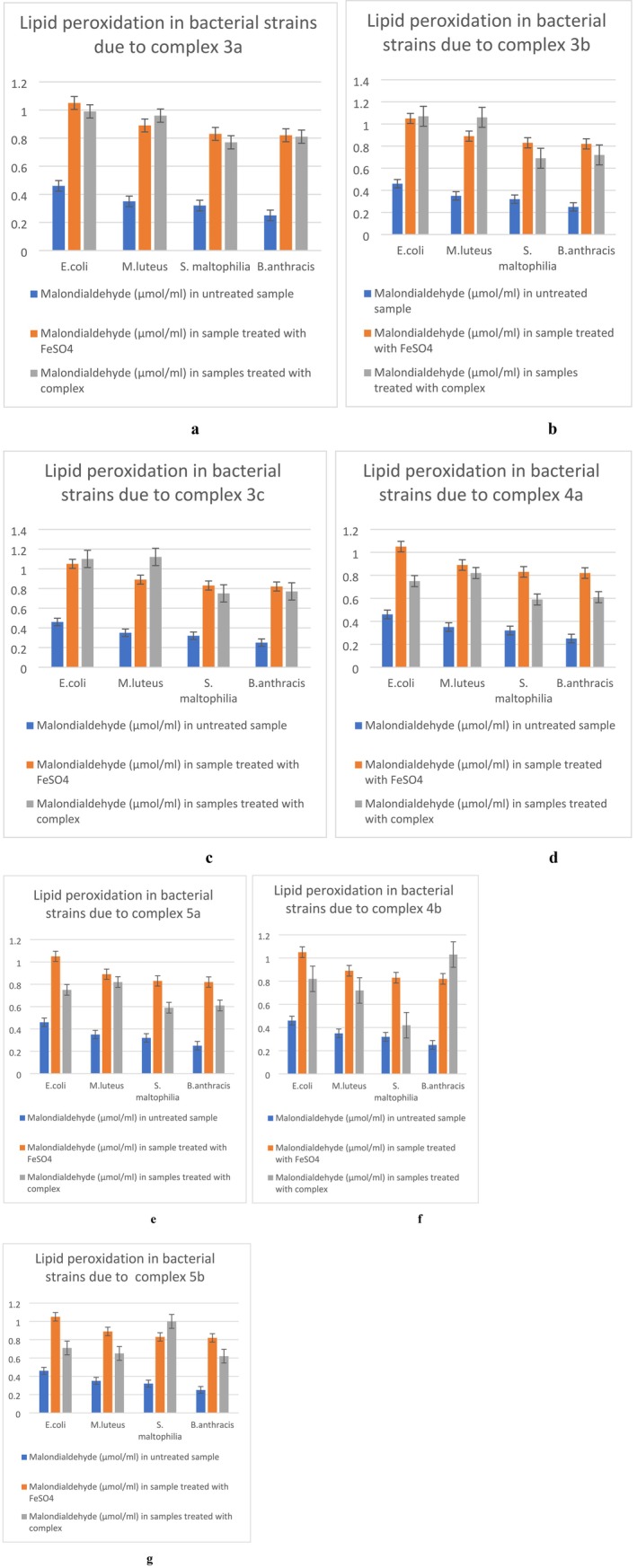
Measurement of MDA concentration, i.e., extent of lipid peroxidation in the bacterial cells by complex 3a (a), complex 3b (b), complex 3c (c), complex 4a (d), complex 5a (e), complex 4b (f), complex 5b (g).

The TBARS assay results have showed that each metallic complex induced the formation of a high amount of Malondialdehyde (MDA) in all four bacterial cultures (Figure 4). This proves that the complexes are causing lipid peroxidation which in turn, probably affects the structural integrity of the bacterial cells. Results of this experiment clearly indicate that the complexes are generating oxidative stress in cells and eventually inhibiting bacterial growth. The concentrations of MDA were measured using the MDA standard curve (Figure S9).

### 3.15 NBT (Nitrobluetetrazolium) Assay to Measure ROS Production

**FIGURE 5:**
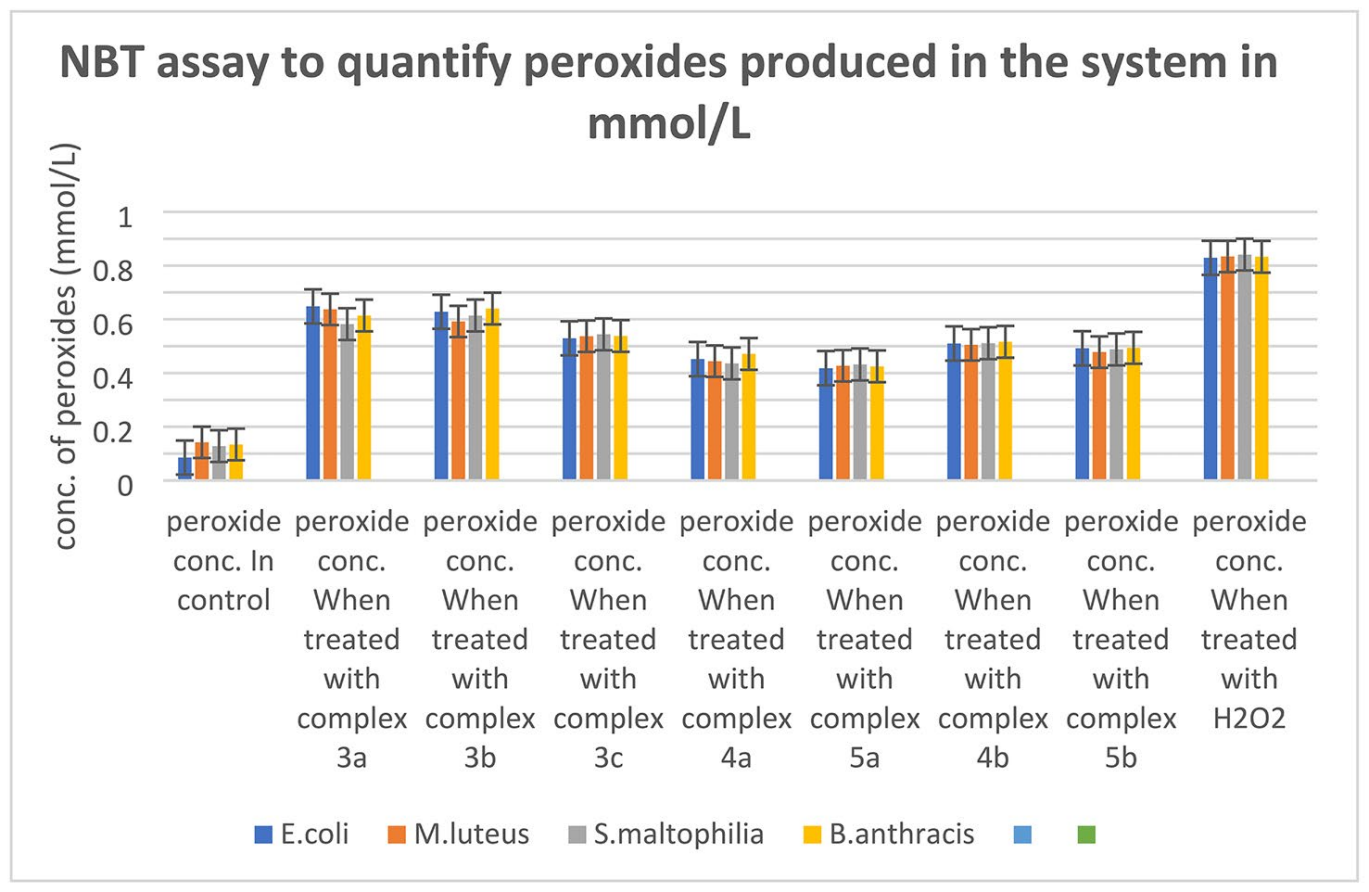
The amount of ROS produced in the microbial cultures treated with the metal complexes confirms the huge oxidative stress generated by the metal complexes.

The results of the NBT assay reveal the production of a high amount of Reactive Oxygen Species (ROS) in the bacterial cells when treated with transition metal complexes (Figure 5 and Table S4). The amount of ROS produced in the microbial cultures treated with the metal complexes confirms the huge oxidative stress generated by the metal complexes. The concentrations of ROS were measured using the standard curve of formazan (Figure S10). The generation of such an amount of ROS and oxidative stress generated by it can cause disruption and/or inhibition of various cellular processes.

### 3.16 Protein and DNA Leakage Assay

**FIGURE 6:**
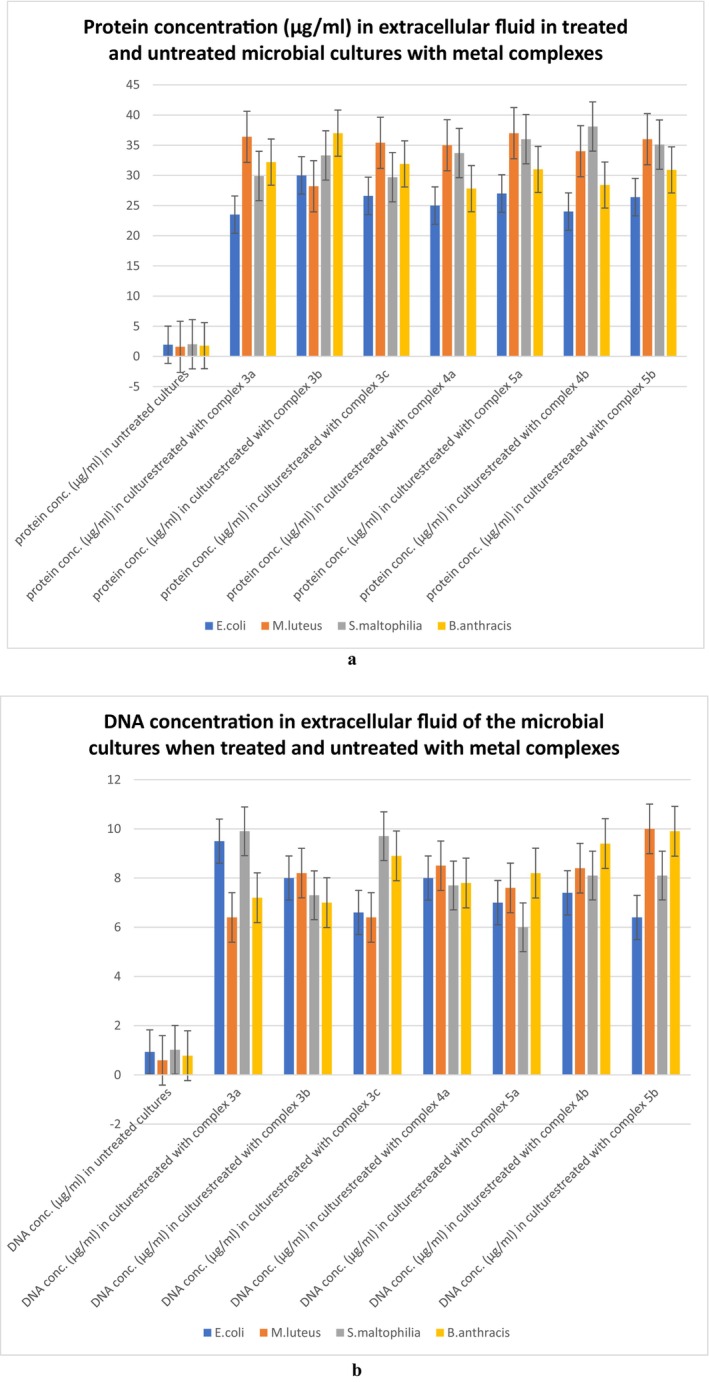
Concentrations of proteins (a) and DNA (b) in extracellular fluid of bacterial cultures after treatment with the metal complexes.

All four bacterial strains have shown significant increments in protein concentration in extracellular fluid when treated with metal complexes (Figure 6a). The metal complex treated cell cultures, when screened for the presence of DNA in the extracellular fluid, exhibited a significant number of nucleic acids (Figure 6b) which were not found in the case of untreated bacterial cultures. The result of this leakage assay strongly points towards membrane damage. The oxidative stress caused by the metal complexes (as mentioned in above experimental results) can be the cause of this membrane damage and subsequent growth reduction.

### 3.17 Measurement of Total Protein Concentration

**FIGURE 7:**
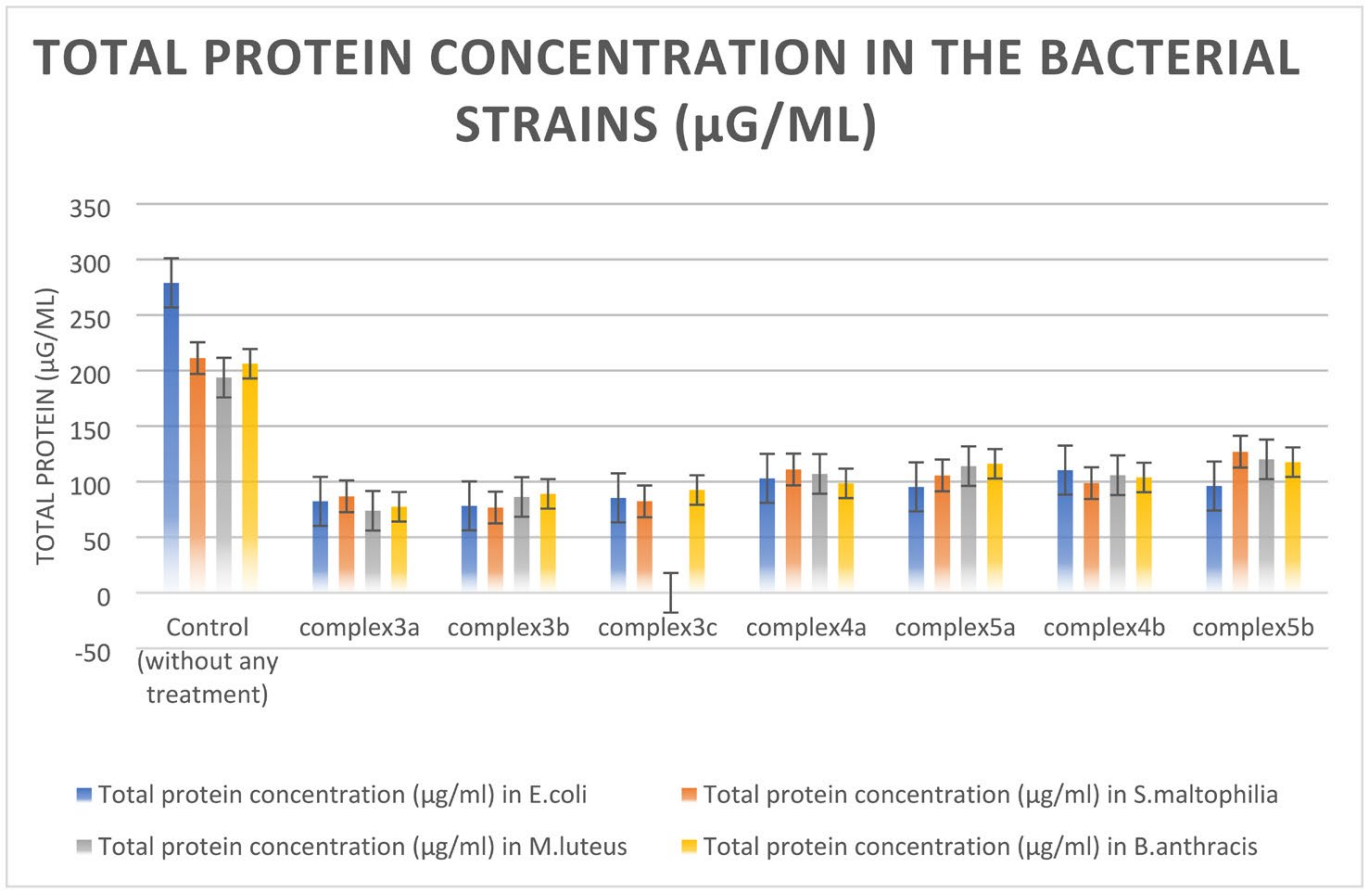
Total protein concentrations (μg/mL) in the bacterial strains with and without the treatment of metal complexes.

A significant amount of reduction in total protein concentrations was observed in all four bacterial strains when treated with the metal complexes (Figure 7 and Table S5). This suggests possible translation inhibition in bacterial cells due to transition metal complexes.

### 3.18 In Vitro Translation Assay

**FIGURE 8:**
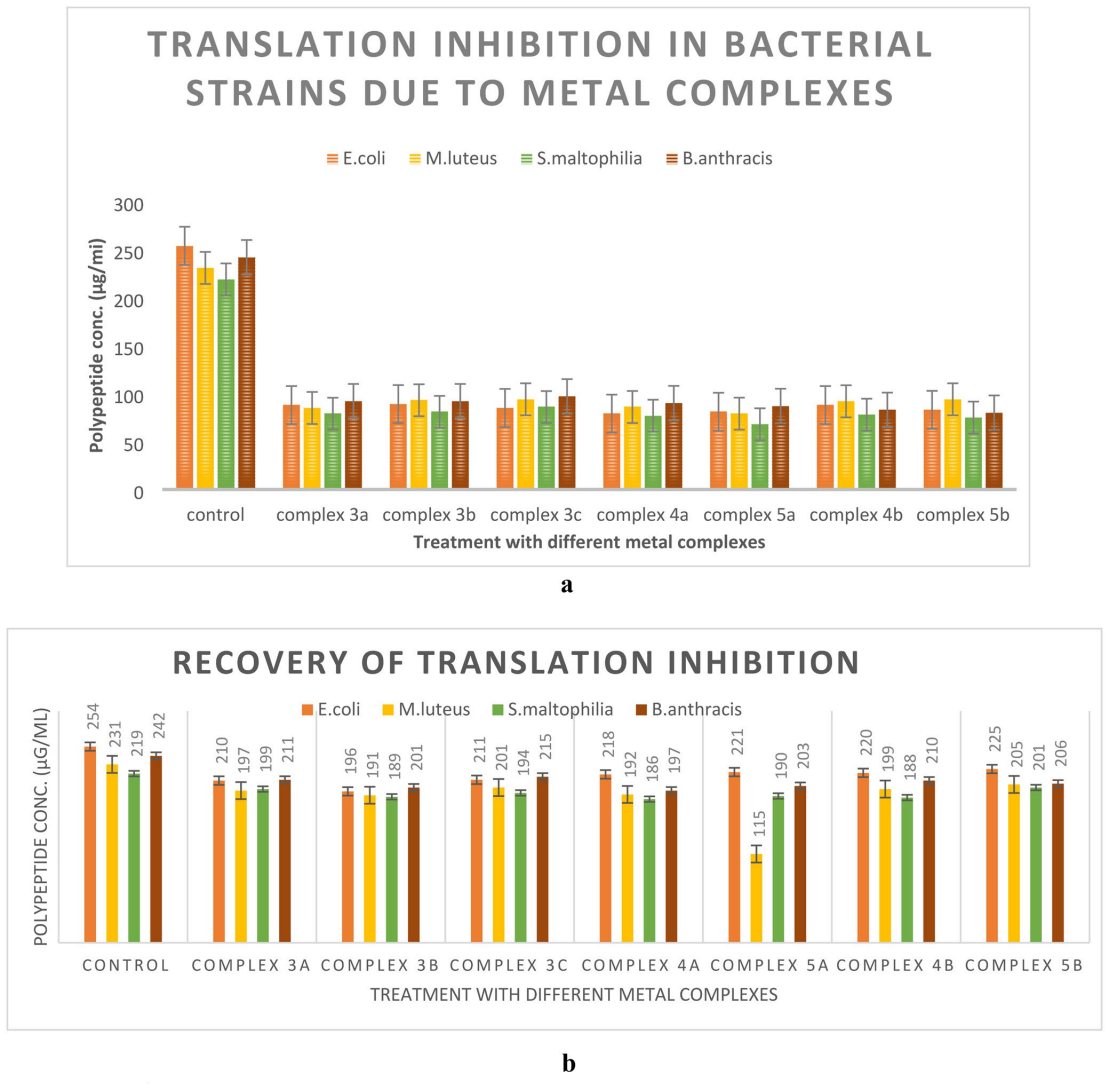
(a) Bacterial strains incubated with metal complexes produced significantly lesser amounts of proteins even in vitro conditions. (b) Recovery of the protein concentration after replacing the metal complex treated total RNA with total RNA from the control set ups.

The results of the in vitro translation assay confirmed that the metal complexes were indeed inhibiting bacterial translation even in in vitro conditions also. The huge difference between the protein concentrations in the reaction mixture containing metal complexes and the mixture without metal complexes made it clear that the metal complexes are blocking the translation process, which resulted in very little protein concentration in the in vitro systems containing metal complexes, in comparison to the control sets without any treatment (Figure 8a). This decline in protein synthesis in the in vitro reaction setups treated with metal complexes was recovered when the total RNA extracted from the treated cells in the test sets was replaced by the same extracted from untreated cell cultures (Figure 8b). This result confirmed that the metal complexes were inhibiting bacterial translation by affecting total RNA. This result correlates with the findings of molecular docking. The probable binding affinity of the metal complexes with the 16S rRNA may indeed cause inhibition of the initiation complex of the bacterial translation process and finally result in translation inhibition.

The result of in vitro translation assay points towards another front of bacterial growth inhibition by the metal complexes apart from the oxidative stress generation as mentioned in afore stated experiment results.

### 3.19 Genomic DNA Extraction and Gel Electrophoresis

**FIGURE 9:**
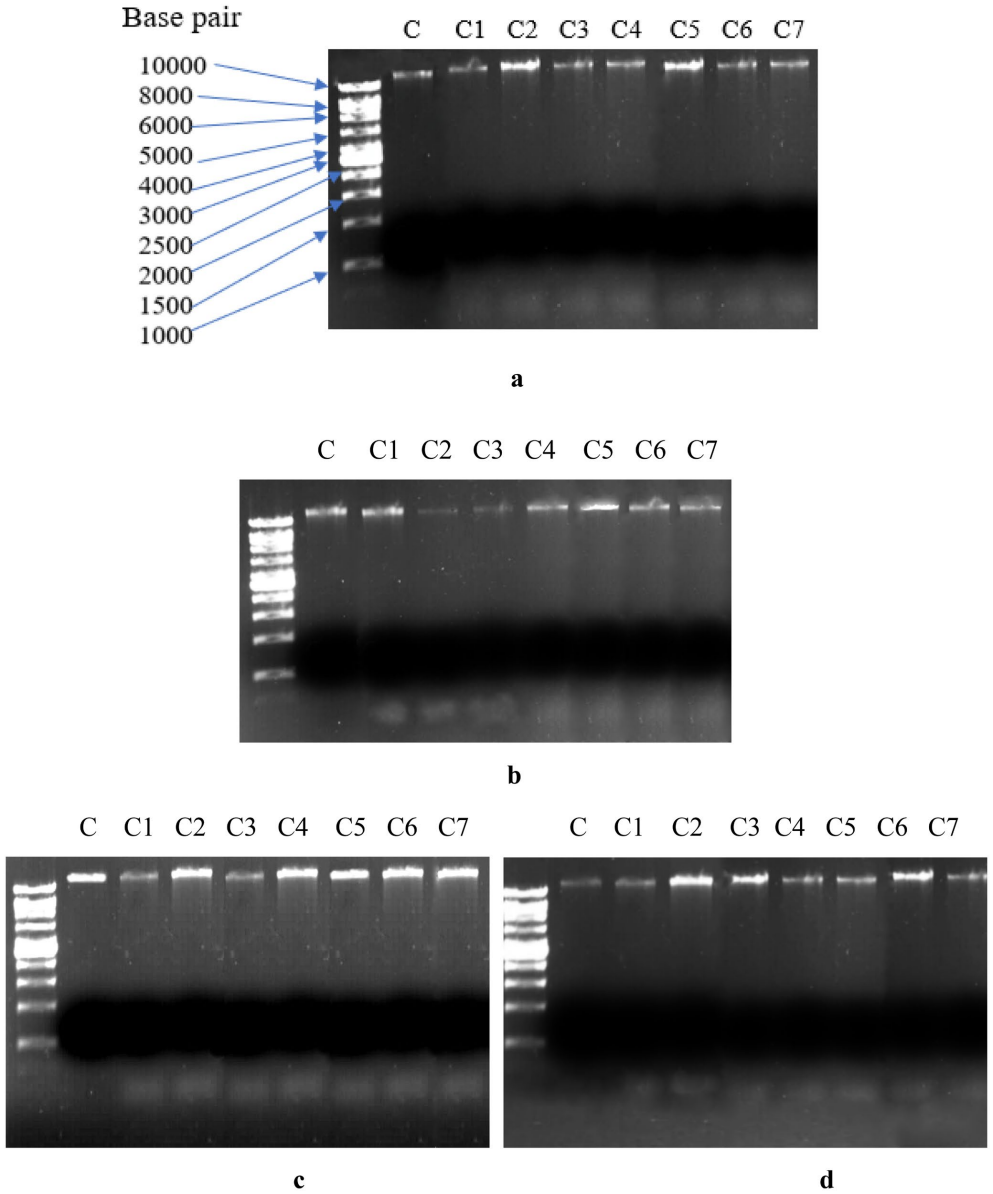
Agarose gel electrophoresis of genomic DNA isolated from *Escherichia coli* (a), *Micrococcus* *luteus* (b), *Stenotrophomonas* *maltophilia* (c), *Bacillus* *anthracis* (d). A separate very light band appeared below the loading dye front in genomic DNA isolated from all four complex‐treated bacterial cultures. In all four pictures, lane C = control (without any treatment), C1 = complex 3a, C2 = complex 3b, C3 = complex 3c, C4 = complex 4a, C5 = complex 5a, C6 = complex 4b, C7 = complex 5b. The same DNA ladder (shown in 9a) has been used in every gel electrophoresis.

Genomic DNA extracted from all four bacterial cultures using a bacterial genomic DNA isolation kit from HiPurA. Both untreated and treated with seven metallic complexes were tested spectrophotometrically (using Nanodrop spectrophotometer Multiscan Sky) for their concentrations. The results showed that in every case, double‐stranded DNA concentration is higher in untreated bacterial cultures than in the cultures treated with metallic complexes (Table 4). This decline in genomic DNA concentration upon incubation with metal complexes suggests the occurrence of DNA damage or inhibition in DNA replication. This conclusion has further been confirmed by the results of DNA agarose gel electrophoresis (Figure 9). The appearance of separate DNA bands in the complex‐treated cells directly points towards DNA damage. However, the bands that appeared even below the loading dye front indicate that those were very small DNA fragments and most probably DNA debris accumulating to form a band.

**TABLE 4:** Concentration of genomic DNA isolated from treated as well as untreated bacterial cultures.

Name of bacterial strains	Concentration of DNA (μg/mL) without any treatment	Concentration of DNA treated with [Fe^III^(Fe^II^(Ph‐L^Ph^)_3_)_2_]ClO_4_ 3a^+^ClO_4_^−^	Concentration of DNA (μg/mL) treated with [Fe^III^(Fe^II^(Py‐L^Ph^)_3_)_2_]ClO_4_ 3b^+^ClO_4_^−^	Concentration of DNA (μg/mL) treated with [Fe^III^(Fe^II^(Np‐L^Ph^)_3_)_2_]ClO_4_ 3c^+^ClO_4_^−^	Concentration of DNA (μg/mL) treated with [Fe^II^(Ph‐L^py^)_2_] 4a	Concentration of DNA (μg/mL) treated with [Mn^II^(Ph‐L^py^)_2_] 5a	Concentration of DNA (μg/mL) treated with [Fe^II^(pTol‐L^py^)_2_] 4b	Concentration of DNA (μg/mL) treated with [Mn^II^(pTol‐L^py^)_2_] 5b
*Escherichia* *coli*	96	63.6	56.2	62.1	58.4	65.5	57.7	67.2
*Stenotrophomonas* *maltophilia*	91.6	55.1	59.2	65	60.5	56	51.8	68.8
*Micrococcus* *luteus*	94.5	59.5	62.4	71.3	58	64.7	66.2	64.8
*Bacillus* *anthracis*	88.7	58.3	51.1	49.2	56.2	63.5	62.9	58

### 3.20 Electrophoretic Mobility Shift Assay (EMSA)

**FIGURE 10:**
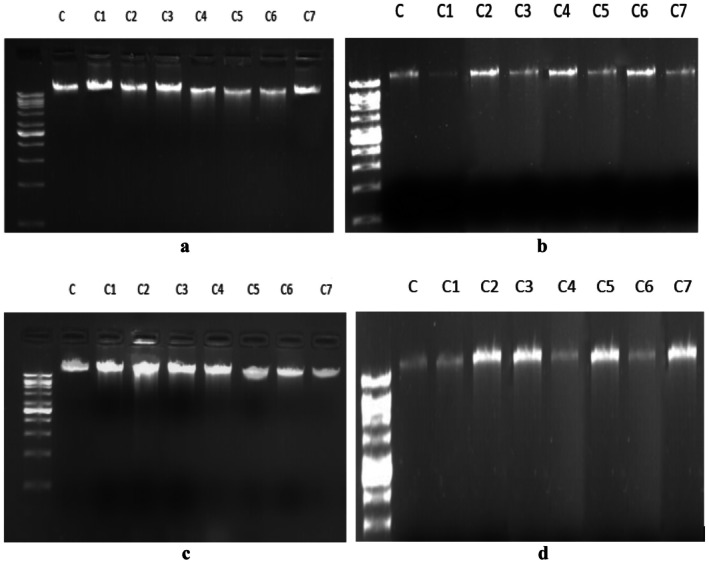
Electrophoretic mobility shift assay of genomic DNA isolated from *Escherichia coli* (a), *Micrococcus* *luteus* (b), *Stenotrophomonas* *maltophilia* (c), *Bacillus* *anthracis* (d) mixed with metal complexes. No significant band shift was found. In all four pictures lane C = control (without any treatment), C1 = complex 3a, C2 = complex 3b, C3 = complex 3c, C4 = complex 4a, C5 = complex 5a, C6 = complex 4b, C7 = complex 5b. The same DNA ladder (shown in Figure 9a) has been used in all four gel electrophoreses.

The genomic DNA isolated from the four bacterial strains when mixed with metal complexes did not cause any band shift in agarose gel electrophoresis (Figure 10), indicating that the metal complexes are not directly binding with the bacterial genomic DNA and causing degradation. The DNA damage by the metal complexes is happening indirectly.

## 4 Discussion

Since bacterial strains in the environment causing dangerous diseases have been genetically evolved to exhibit defence against successful as well as frequently used antibiotics, the need for different kinds of therapeutic drugs to address this issue has become evident. The recent research findings revealed that inorganic metallic complexes are quite fruitful in this regard.

**FIGURE 11:**
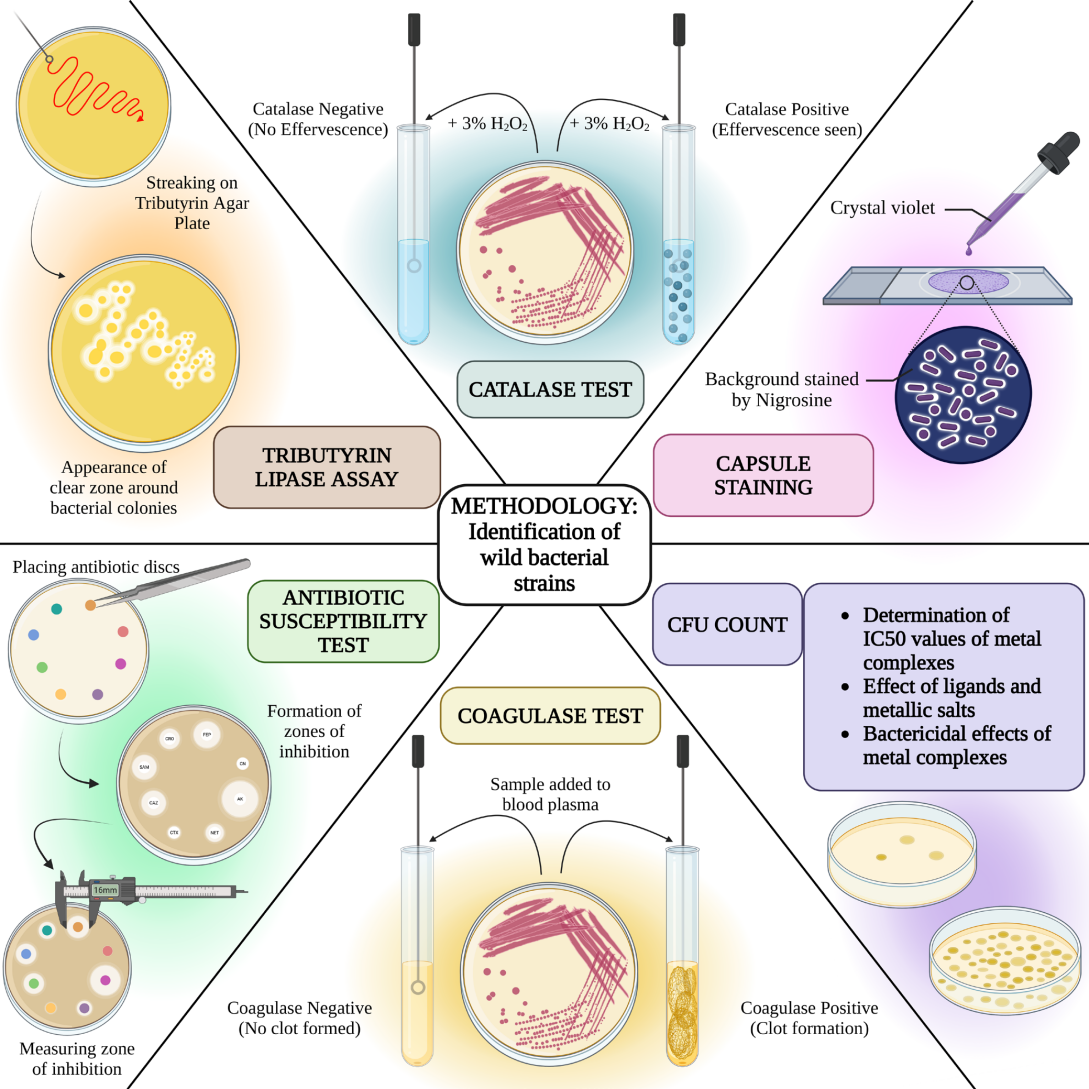
Schematic representation of performed biochemical characterisation of isolated bacterial strains.

These inorganic metal complexes, when applied in biological fields, are tested mostly on isolated, pre‐characterised pure cultures of bacterial strains to determine their activity against those specific organisms. In this project, the antibacterial agents synthesised have been applied to the natural sources of MDRs (sewage water near hospital compound) in a wholistic manner and achieved satisfactory results. Multiple experiments uncovered that the most abundant bacterial strains present in the sample were resistant to multiple broad‐spectrum antibiotics like Vancomycin, Clindamycin, Aztreonam, Oxacillin, Cephalothin, Erythromycin, Amoxyclav, Ceftazidime, etc. The 16srRNA analysis confirmed the identity of four bacterial strains (*E. coli, S. maltophilia, M. luteus, B. anthracis*) that were used for further research. Moreover, pathogenicity analysis performed revealed that these bacterial strains present in that sample have a high probability of being human pathogens. Like *E. coli* (causative agents for urinary tract infection), *B. anthracis* (causative agent for anthrax [32]), *S. maltophilia* (causative agent for (haematological disease, organ disfunction, nosocomial infections [33, 34])), *M. luteus* (causative agent for rhabdomyosarcoma, brain abcess [35, 36]). All these bacterial strains were able to produce catalase as well as coagulase (except *S. maltophilia*) which further points towards their probable pathogenicity. *S. maltophilia* producing lipase is normally found to be pathogenic [37]. Hence, the strain isolated in this project is most probably pathogenic. In *B. anthracis*, the virulent gene is closely associated with the capsule‐producing gene [37], hence the presence of the capsule confirms the presence of pathogenicity of the bacterial strain. In most research on this topic, laboratory‐made pure cultures of the aforesaid bacterial strains have been used, but pathogenic MDRs in natural samples successfully inhibited by azo‐oxime metal complexes are a very promising indication towards the eradication of MDRs from water (Figure 11).

**FIGURE 12:**
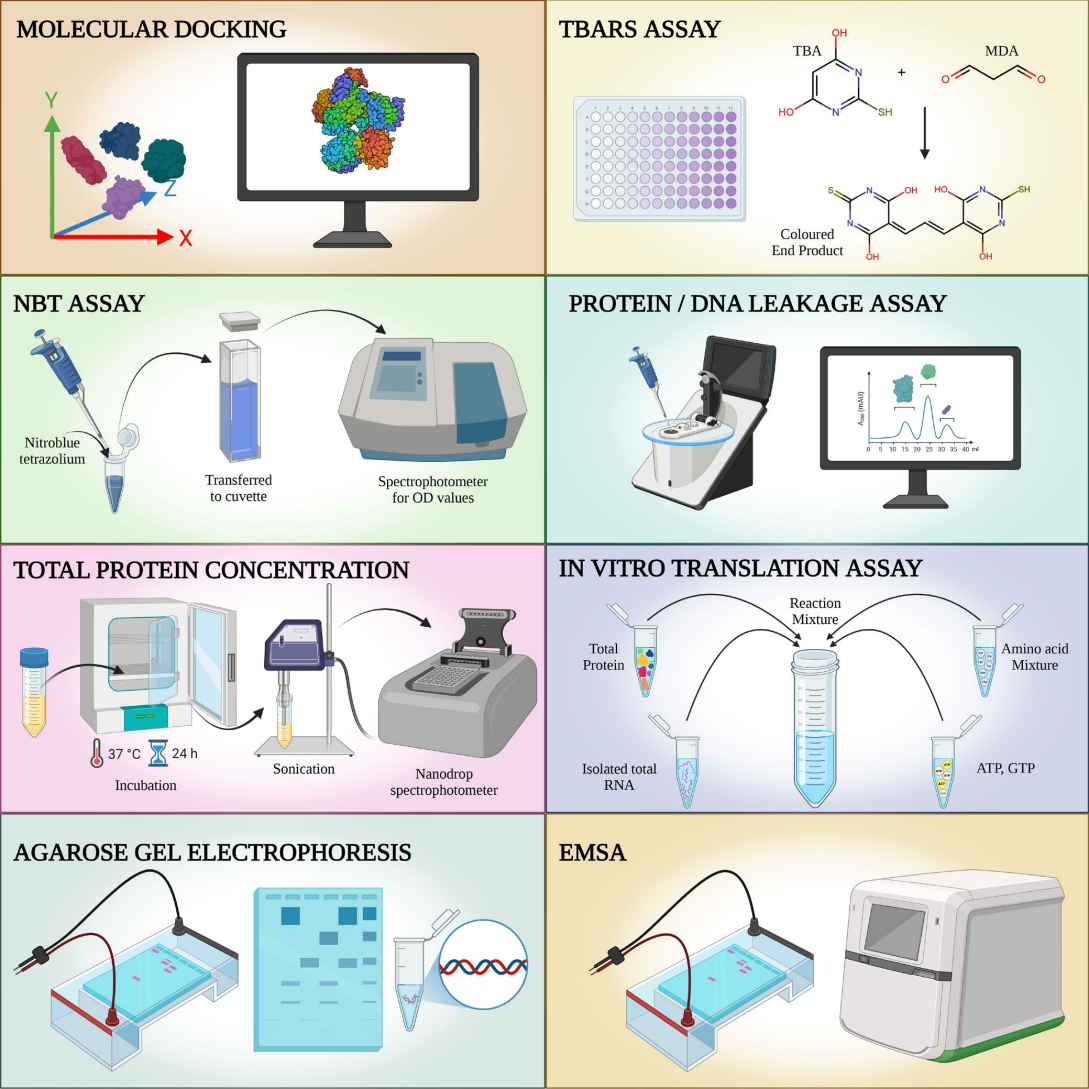
Schematic representation of the molecular mode of action and assays involved in the project.

In this project, the TBARS (or lipid peroxidation) assay and NBT assay results confirmed the production of significant amounts of ROS (reactive oxygen species) which are in turn damaging cell membranes (bacterial cell membrane contains lipid layers, hence most abundant source of lipid in cellular environment, peroxidation of lipid molecules in membrane will definitely cause membrane damage) [38] and hence permitting the leakage of intracellular proteins as well as nucleic acids in extracellular fluid. These damaged cell membranes in all four bacterial cultures treated by metal complexes are definitely aiding cell death. A decrease in total protein concentrations in complex‐treated bacterial cultures points towards translation inhibition, which is predicted by molecular docking, as it suggested high binding affinity of the metal complexes with the 30S ribosome subunit near the Shine Dalgarno sequence, which could inhibit translation initiation. The results of the in vitro translation assay point towards damage or non‐functionality of RNA, as the translation process was recovered after the total RNA isolated from the treated cells was replaced by the total RNA isolated from untreated bacterial cultures. But we could not specify physically if the metal complexes were actually binding with the 30S ribosomal subunit from this assay. The reduction in DNA concentration in the bacterial cultures treated by the metal complexes can be the result of deactivated DNA gyrase [39], if the metal complexes bind to the enzyme as suggested by the molecular docking data. The separate DNA band that appeared in the DNA agarose gel electrophoresis is not the direct effect of the metal complexes, as these metal complexes cannot bind with the bacterial genomic DNA, which is established by the EMSA (electrophoretic mobility shift assay) test results. Hence, the DNA debris (the bands appeared even below the dye fronts, hence these are very small DNA debris) are not directly produced by the metal complexes, but they can be the result of ROS generated by the metal complexes [40]. Antibiotic resistance occurs due to genetic modifications in bacterial strains that alter cellular systems previously targeted by antibacterial agents. Hence, simpler processes of inhibition are more susceptible to genetic evolution. Silver nanoparticles have been revealed to obstruct the growth of *E. coli* by either degrading their cell membrane through oxidative stress or, in some instances, through DNA degeneration. Nevertheless, silver‐tolerant bacteria have also been located (e.g., *P. stutzeri*, *K. pneumonae* etc.) [41], which proved the necessity of synthesising antibacterial agents with multiple modes of action (Figure 12), so that to overcome the stress caused by antibiotics, the bacterial strains have to evolve in multiple cellular mechanisms, which is not very easily done. The antibacterial agents synthesised in this project have shown activities on multiple fronts, such as lipid peroxidation and subsequent membrane damage, as well as DNA damage and translation inhibition by disturbing the RNAs (most probably 30S ribosome subunit, as predicted by molecular docking). The versatility of the modes of action of the metal complexes makes them more potent antibacterial agents than many other antibacterial drugs.

## 5 Conclusion

In this project we have focussed on elucidating a sustainable answer to the threat caused by immensely escalated antibiotic resistance in bacterial strains with Fe(II) and Mn(II) complexes of azo‐oximate frameworks in natural conditions. Tests have been performed on wild bacterial populations from one of the most common sources of MDRs, sewage water near a hospital compound. All seven complexes synthesised have successfully inhibited bacterial growth in a wholistic manner, which is confirmed by reduced colony count. Further investigation about the nature of the bacterial strains present in the water sample revealed that the most abundant bacterial colonies (*E. coli, S. maltophilia, M. luteus, B. anthracis*) present in the water sample were not only pathogenic, but also resistant to multiple broad‐spectrum antibiotics like Vancomycin, Amoxiclav, etc. The experiments on the molecular mechanism of action of the complexes revealed that the complexes are inhibiting bacterial growth by effecting multiple domains in bacterial strains. By producing a high amount of ROS, the metal complexes are creating huge oxidative stress in the bacterial cellular environment and subsequently causing lipid peroxidation, hence leading to membrane damage and producing DNA debris by DNA damage. Translation machinery was also affected by the metal complexes through inactivation of RNAs. The complexes with Fe(II) seemed to be more potent than Mn(II) complexes. The synthesis process of these complexes is simple, less time‐taking, economically reasonable and reagents are easily available. Moreover, Fe and Mn, being available naturally in biological systems, are less toxic at low concentrations. Hence, in summary, it can be concluded that the Fe and Mn azo‐oximates may act as tools to find the answer to sustainably eradicate MDRs from the water sources.

## Author Contributions

**Aratrika Samajdar:** conceptualization (equal), data curation (equal), formal analysis (equal), investigation (equal), methodology (equal), software (equal), validation (equal), visualization (equal), writing – original draft (equal). **Supriyo Halder:** conceptualization (equal), data curation (equal), formal analysis (equal), investigation (equal), methodology (equal), software (equal), validation (equal), writing – original draft (equal). **Sukanya Chatterjee:** data curation (equal), formal analysis (equal), investigation (equal), methodology (equal), software (equal), validation (equal), writing – original draft (equal). **Debjeet Chakraborty:** data curation (equal), formal analysis (equal), investigation (equal), methodology (equal), software (equal), validation (equal), writing – original draft (equal). **Arup Kumar Mitra:** formal analysis (equal), investigation (equal), methodology (equal), project administration (equal), supervision (equal), validation (equal), visualization (equal), writing – review and editing (equal). **Anindita Banerjee:** conceptualization (equal), formal analysis (equal), investigation (equal), project administration (equal), supervision (equal), validation (equal), visualization (equal), writing – review and editing (equal). **Kausikisankar Pramanik:** conceptualization (equal), data curation (equal), formal analysis (equal), funding acquisition (equal), project administration (equal), resources (equal), supervision (equal), validation (equal), visualization (equal), writing – review and editing (equal). **Sanjib Ganguly:** conceptualization (equal), data curation (equal), formal analysis (equal), funding acquisition (equal), investigation (equal), methodology (equal), project administration (equal), resources (equal), supervision (equal), validation (equal), visualization (equal), writing – review and editing (equal). **Ajoy Kumer:** conceptualization (equal), funding acquisition (equal), investigation (equal), project administration (equal), software (equal), supervision (equal), validation (equal), visualization (equal), writing – review and editing (equal). **Bikram Dhara:** formal analysis (equal), funding acquisition (equal), investigation (equal), methodology (equal), software (equal), supervision (equal), validation (equal), visualization (equal), writing – review and editing (equal).

## Consent

The authors have nothing to report.

## Conflicts of Interest

The authors declare no conflicts of interest.

## Supporting information


**Figure S1:** Bacterial growth from sewage water on nutrient agar plates containing complex 3a (b), complex 3b (c), complex 3c (d), complex 4a (e), complex 5a (f), complex 4b (g), complex 5b (h). Figure 1a represents bacterial colonies from sewage water with 10^−3^ dilution on nutrient agar plate without any metallic complex.
**Table S1:** Numbers of bacterial colonies from sewage water on nutrient agar plates treated as well as untreated with metallic complexes.
**Figure S2:** Figure 5b–e represent results of catalase test for bacterial strain *E. coli, S. maltophilia, M. luteus* and *B. anthracis* respectively, where as 5a represents the result of exposing catalase negative *Streptococcus* sp. to H_2_O_2_. The presence of bubbles in the test tubes (5b–e) indicates that all four bacterial cultures isolated from sewage water sample can produce catalase in presence of oxidative stress.
**Figure S3:** (a–d) represent the results of incubating *S. maltophilia*, *E. coli, M. luteus* and *B. anthracis* respectively, in goat blood plasma. (e) represents the control set where no bacterial culture were added to the plasma sample. The opaque substance present in three test sets (b–d) proves the ability of the bacterial cultures to produce coagulase enzyme, where as *S. maltophilia* (a) did not form any opaque substance indicating that this bacterial strain does not produce coagulase.
**Figure S4:** Antibiotic susceptibility of *B. anthracis* (a, b); Antibiotic susceptibility of *S. maltophilia* (c, d); Antibiotic susceptibility of *E. coli* (e, f); Antibiotic susceptibility of *M. luteus* (g, h); AK = Amikacin; AMC = Amoxiclav; AT = Aztreonam; CAZ = Ceftazidine; CD = Clindamycin; CEP = Cephalothin; CTX = Cefotaxime; E = Erythromycin; IPM = Imipenem; LE = Levofloxacin; OX = Oxacillin; *P* = Penicillin G.
**Figure S5:** Growth of *S. maltophilia* on tributyrin agar. The clear zone around the bacterial colonies indicated lipase production.
**Table S2a:** Growth reduction of *E. coli* by the metal complexes, measured by CFU count.
**Table S2b:** Growth reduction of *S. maltophilia* by the metal complexes, measured by CFU count.
**Table S2c:** Growth reduction of *M. luteus* by the metal complexes, measured by CFU count.
**Table S2d:** Growth reduction of *B. anthracis* by the metal complexes, measured by CFU count.
**Figure S6:** Growth inhibition of *M. luteus* by ligand 1b compared to its Fe complex is nominal in compared to the respective metal complex.
**Figure S7a:** Microbial growth inhibition by Fe(II) perchlorate salt compared to respective complexes.
**Figure S7b:** Microbial growth inhibition by Mn(II) salt compared to respective complexes.
**Figure S8:** Capsule staining of *B. anthracis*. The halo zone surrounding the cells represents capsule.
**Table S3:** Observation of the bactericidal effect all the seven metal complexes (a–g represents the results of complex 3a, 3b, 3c, 4a, 4b, 5a, 5b respectively) on four bacterial strains by using CFU counts.
**Figure S9:** MDA standard curve.
**Figure S10:** Formazan standard curve.
**Table S4:** Amount (mmol/L) of ROS produced by different metal complexes in different bacterial strains.
**Table S5:** Total protein concentrations of all the four bacterial strains with and without the treatment with matal complexes.
**Figure S11a:** Binding interactions of complex 5a (highlighted in magenta) and chain A of DNA Gyrase (PDB: 6RKS) subunit of *Escherichia coli* forming H bonds (shown in red) with LYS 298 (H bond restraint 0.4 Å and 120°).
**Figure S11b:** Binding interactions of complex 5b (highlighted in magenta) and chain B and chain C of DNA Gyrase (PDB: 6RKS) subunit of *Escherichia coli* forming H bonds (shown in red) with ASN 437 and LYS 298 respectively (H bond restraint 0.4 Å and 120°).
**Figure S11c:** Binding interactions of complex 4a (highlighted in magenta) and Chain D of DNA Gyrase (PDB: 6RKS) of *Escherichia coli* forming H bonds (shown in red) with GLN 434 (H bond restraint 0.4 Å and 20°).
**Figure S11d:** Binding interactions of complex 4b (highlighted in magenta) and Chain D of DNA Gyrase (PDB: 6RKS) of *Escherichia coli* forming H bonds (shown in red) with GLN 434 (H bond restraint 0.4 Å and 20°).
**Table S6:** Normality test of IC50 values of different bacterial strains by the metal complexes using Shapiro Wilks method.

## Data Availability

All the data used are available within the manuscript and in the Supporting Information.
