# Supplementary material for: Iron and Manganese Azooxime Complexes as Anti‐Microbial Agents Against Antibiotics Resistant Wild Bacteria From Hospital Drainage
Source: J Cell Mol Med. 2025 Sep 5;29(17):e70826. doi: 10.1111/jcmm.70826 (PMC12413315; doi:10.1111/jcmm.70826)
Supplement: Supplementary file 1 — Figure S1: Bacterial growth from sewage water on nutrient agar plates containing complex 3a (b), complex 3b (c), complex 3c (d), complex 4a (e), complex 5a (f), complex 4b (g), complex 5b (h). Figure 1a represents bacterial colonies from sewage water with 10−3 dilution on nutrient agar plate without any metallic complex. Table S1: Numbers of bacterial colonies from sewage water on nutrient agar plates treated as well as untreated with metallic complexes. Figure S2: Figure 5b–e represent results of catalase test for bacterial strain E. coli, S. maltophilia, M. luteus and B. anthracis respectively, where as 5a represents the result of exposing catalase negative Streptococcus sp. to H2O2. The presence of bubbles in the test tubes (5b–e) indicates that all four bacterial cultures isolated from sewage water sample can produce catalase in presence of oxidative stress. Figure S3: (a–d) represent the results of incubating S. maltophilia, E. coli, M. luteus and B. anthracis respectively, in goat blood plasma. (e) represents the control set where no bacterial culture were added to the plasma sample. The opaque substance present in three test sets (b–d) proves the ability of the bacterial cultures to produce coagulase enzyme, where as S. maltophilia (a) did not form any opaque substance indicating that this bacterial strain does not produce coagulase. Figure S4: Antibiotic susceptibility of B. anthracis (a, b); Antibiotic susceptibility of S. maltophilia (c, d); Antibiotic susceptibility of E. coli (e, f); Antibiotic susceptibility of M. luteus (g, h); AK = Amikacin; AMC = Amoxiclav; AT = Aztreonam; CAZ = Ceftazidine; CD = Clindamycin; CEP = Cephalothin; CTX = Cefotaxime; E = Erythromycin; IPM = Imipenem; LE = Levofloxacin; OX = Oxacillin; P = Penicillin G. Figure S5: Growth of S. maltophilia on tributyrin agar. The clear zone around the bacterial colonies indicated lipase production. Table S2a: Growth reduction of E. coli by the metal complexes, measured by CFU count. Table S [file JCMM-29-e70826-s001.docx]

**Iron and Manganese Azooxime Complexes as Anti-Microbial Agents Against Antibiotics Resistant Wild Bacteria from Hospital Drainage**

*Aratrika Samajdar^a^, Supriyo Halder^b^, Sukanya Chatterjee^a^, Debjeet Chakraborty^a^, Arup Kumar Mitra^*a^, Anindita Banerjee^a^, Kausikisankar Pramanik^b^, Sanjib Ganguly^c^, Ajoy Kumer*^d^, Bikram Dhara*^e^*

**^a^**Department of Microbiology St. Xavier’s College (Autonomous), 30, Park Street, Kolkata- 700016, Kolkata, India. ([aratrika7521@gmail.com](mailto:aratrika7521@gmail.com) ; [chatterjee092024@gmail.com](mailto:chatterjee092024@gmail.com) ; [debjeetchakraborty44@gmail.com](mailto:debjeetchakraborty44@gmail.com) ; [drakmitra01@sxccal.edu](mailto:drakmitra01@sxccal.edu) ; [anni79in@gmail.com](mailto:anni79in@gmail.com))

^b^Department of Chemistry, Jadavpur University, Kolkata 700032, Kolkata, India. (SH: [supriyochem93@gmail.com](mailto:supriyochem93@gmail.com) ; [kpramanik@hotmail.com](mailto:kpramanik@hotmail.com))

^c^Department of Chemistry St. Xavier’s College (Autonomous), 30, Park Street, Kolkata- 700016, Kolkata, India. ([icsg@sxccal.edu](mailto:icsg@sxccal.edu))

^d^Department of Chemistry, IUBAT-International University of Business Agriculture & Technology, Embankment Drive Road, Dhaka 1230, Bangladesh. ([kumarajoy.cu@gmail.com](mailto:kumarajoy.cu@gmail.com))

^e^Centre for Global Health Research, Saveetha Medical College and Hospital, Saveetha Institute of Medical and Technical Sciences, India. ([bikramdhara.smc@saveetha.com](mailto:bikramdhara.smc@saveetha.com))

***Corresponding Author:** Arup Kumar Mitra ([drakmitra01@sxccal.edu](mailto:drakmitra01@sxccal.edu)), Ajoy Kumer ([kumarajoy.cu@gmail.com](mailto:kumarajoy.cu@gmail.com)). Bikram Dhara ([bikramdhara.smc@saveetha.com](mailto:bikramdhara.smc@saveetha.com)).


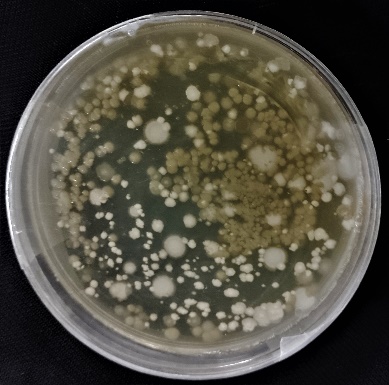

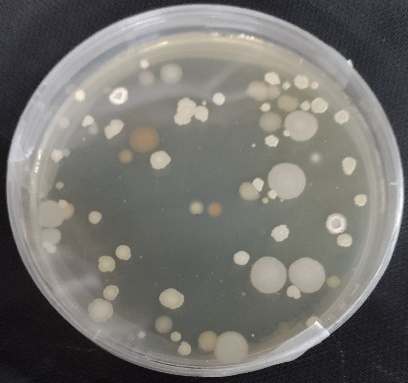

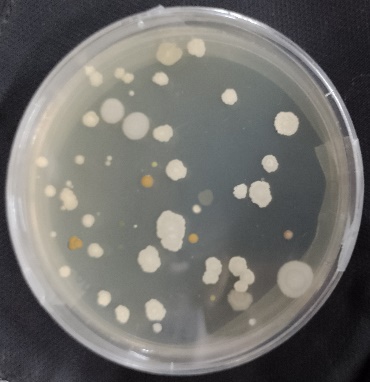


1a 1b 1c


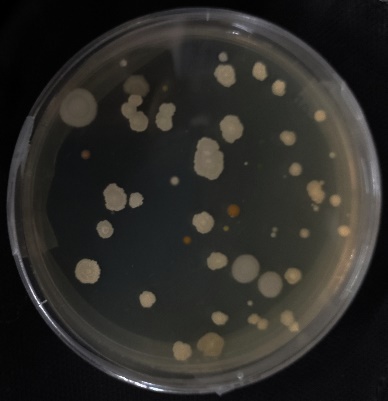

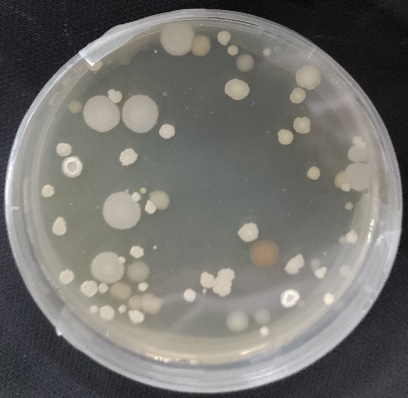

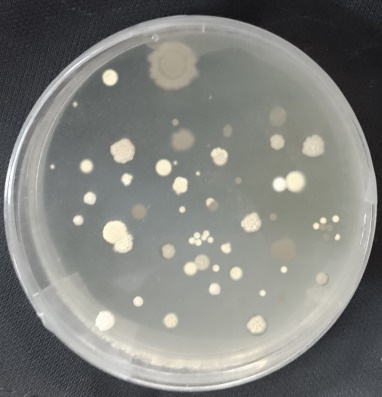


1d 1e 1f


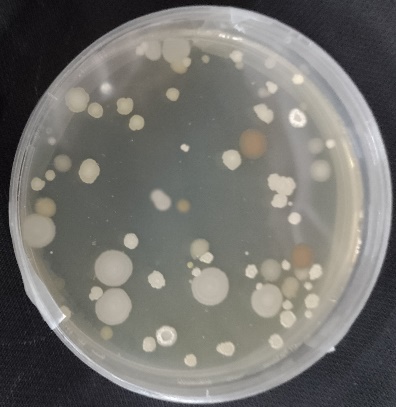

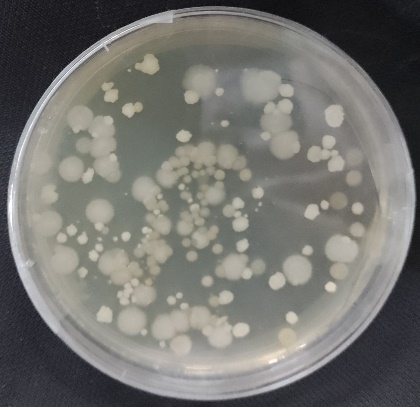


1g 1h

**Figure S1:** Bacterial growth from sewage water on nutrient agar plates containing complex 3a (1b), complex 3b (1c), complex 3c (1d), complex 4a (1e), complex 5a (1f), complex 4b (1g), complex 5b (1h). figure 1a represents bacterial colonies from sewage water with 10^-3^ dilution on nutrient agar plate without any metallic complex.

| **Name of complex** | **CFU in 10^-3^ dilution** |
| --- | --- |
| **CONTROL** | 1250 |
| **Complex 3a** | 87 |
| **Complex 3b** | 63 |
| **Complex 3c** | 68 |
| **Complex 4a** | 72 |
| **Complex 5a** | 112 |
| **Complex 4b** | 90 |
| **Complex 5b** | 220 |

**Table S1:** Numbers of bacterial colonies from sewage water on nutrient agar plates treated as well as untreated with metallic complexes.


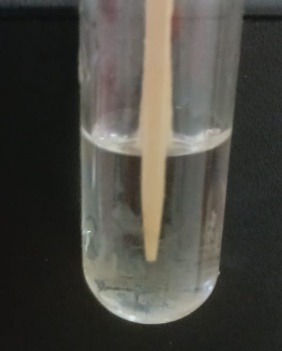

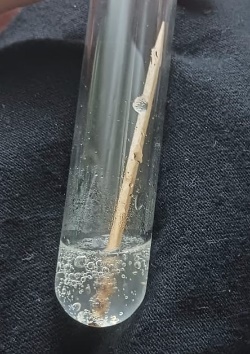

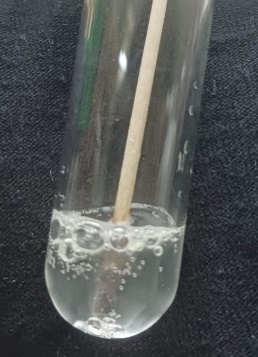

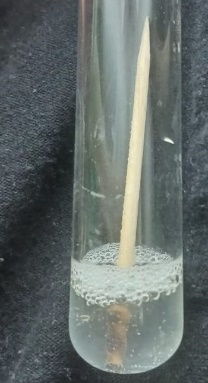

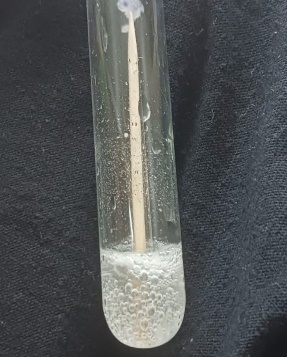


5a 5b 5c 5d 5e

**Figure S2:** Figure 5b to 5e represent results of catalase test for bacterial strain *E.coli, S.maltophilia, M.luteus* and *B.anthracis* respectively, where as 5a represents the result of exposing catalase negative *Streptococcus.sp* to H_2_O_2._ The presence of bubbles in the test tubes (5b-5e) indicates that all four bacterial cultures isolated from sewage water sample can produce catalase in presence of oxidative stress.


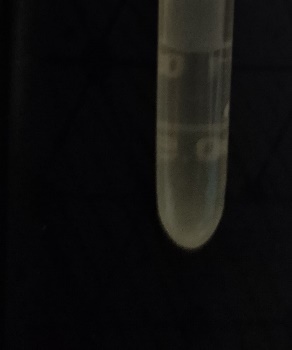

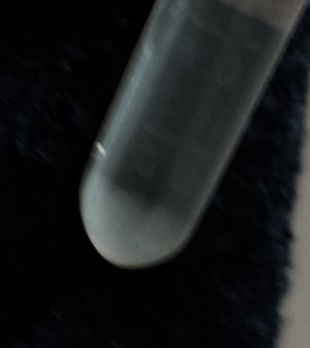

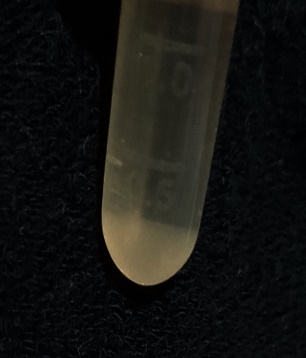

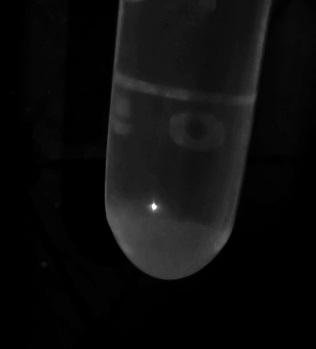


S3a S3b S3c S3d


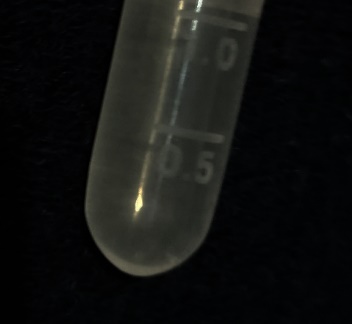


S3e

**Figure S3:** Figure S3a to S3d represent the results of incubating *S.maltophilia* *E.coli, , M.luteus* and *B.anthracis* respectively, in goat blood plasma. Figure S3e represents the control set where no bacterial culture were added to the plasma sample. The opaque substance present in three test sets (S3b to S3d) proves the ability of the bacterial cultures to produce coagulase enzyme, where as *S.maltophilia* (S3a) did not form any opaque substance indicating that this bacterial strain does not produce coagulase.


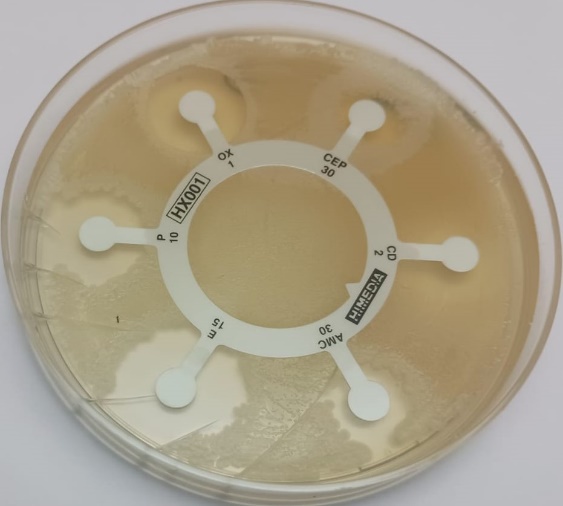

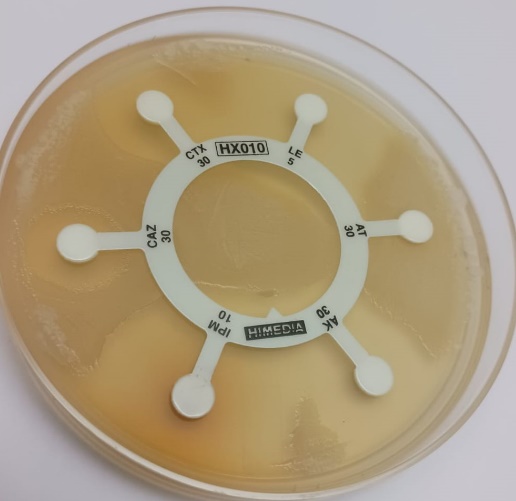


S4a S4b


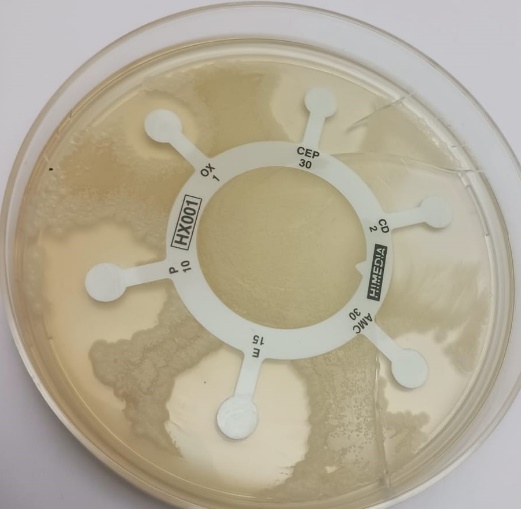

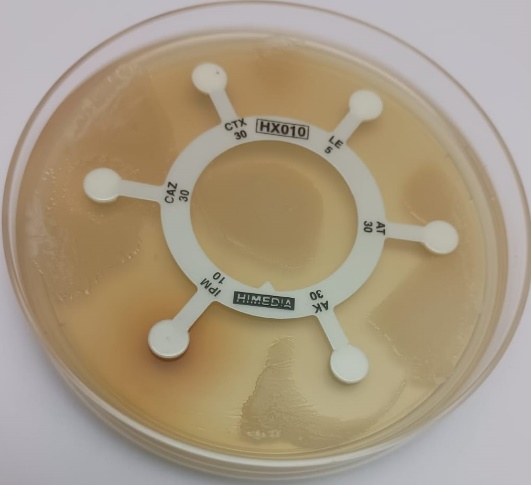


S4c S4d


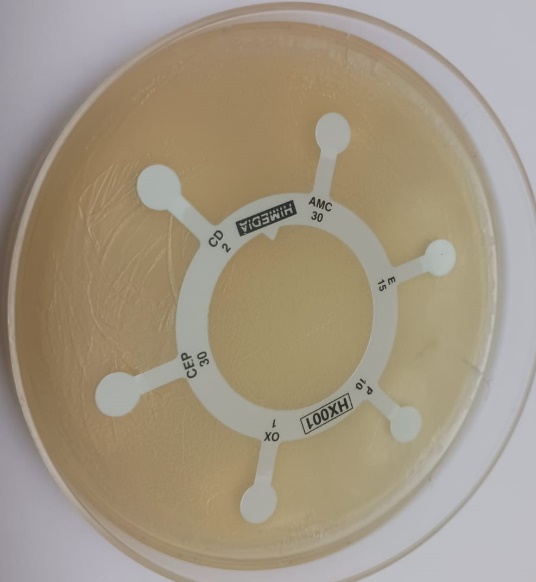

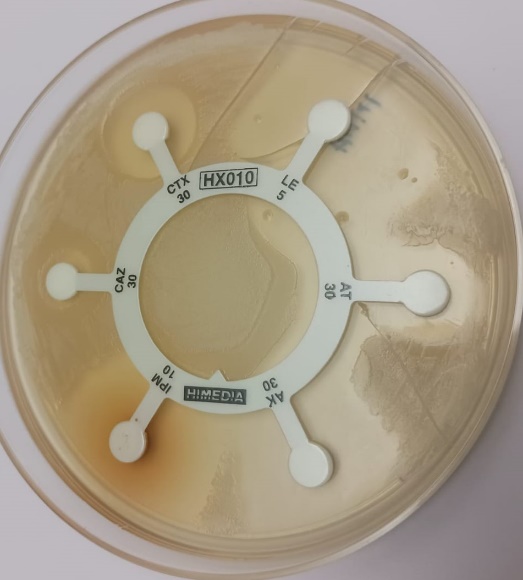


S4e S4f


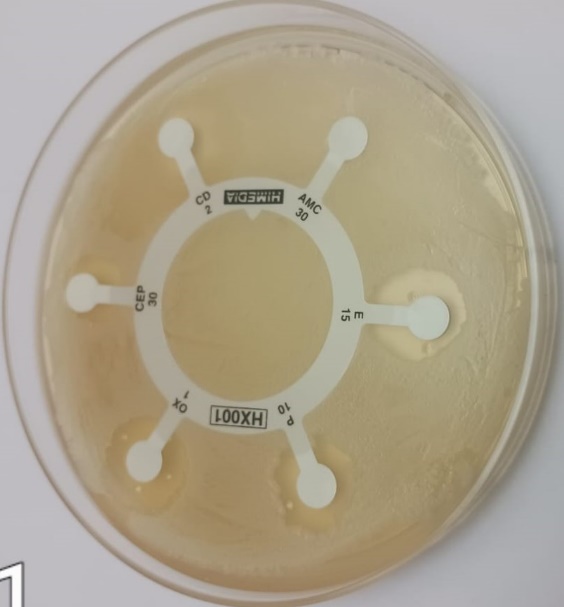

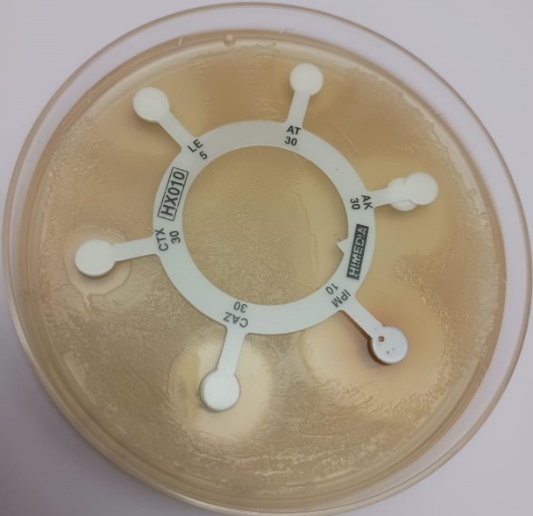


S4g S4h

**Figure S4:** Antibiotic susceptibility of *B.anthracis* (S4a, S4b); Antibiotic susceptibility of *S.maltophilia* (S4c, S4d); Antibiotic susceptibility of *E.coli* (S4e, S4f); Antibiotic susceptibility of *M.luteus* (S4g, S4h); CTX= Cefotaxime, LE= Levofloxacin, AT= Aztreonam, IPM= Imipenem, AK= Amikacin, CAZ= Ceftazidine, P= Penicillin G, OX= Oxacillin, CEP= Cephalothin, CD= Clindamycin, E= Erythromycin, AMC= Amoxiclav.


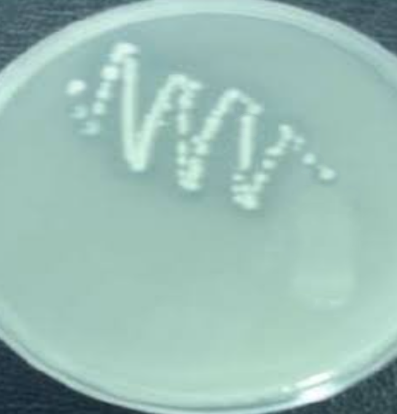


**Figure S5:** Growth of *S.maltophilia* on tributyrin agar. The clear zone around the bacterial colonies indicated lipase production.

| Strength of treatment | CFU/ml in control (without treatment) | CFU/ml in solvent control (with 100ppm DCM) | CFU/ml when treated with Complex 3a | CFU/ml when treated with Complex 3b | CFU/ml when treated with Complex 3c | CFU/ml when treated with Complex 4a | CFU/ml when treated with Complex 5a | CFU/ml when treated with Complex 4b | CFU/ml when treated with Complex 5b | CFU/ml when treated with Tetracycline |
| --- | --- | --- | --- | --- | --- | --- | --- | --- | --- | --- |
| 10ppm | 5.83 x 10^6^ | 4.88 x 10^6^ | 4.39 x 10^6^ | 4.16 x 10^6^ | 4.04 x 10^6^ | 4.30 x 10^6^ | 4.17 x 10^6^ | 4.11 x 10^6^ | 4.15 x 10^6^ | 4.30 x 10^6^ |
| 30ppm | 5.83 x 10^6^ | 4.88 x 10^6^ | 3.57 x 10^6^ | 3.69 x 10^6^ | 3.34 x 10^6^ | 3.55 x 10^6^ | 3.40 x 10^6^ | 3.37 x 10^6^ | 3.64 x 10^6^ | 3.61 x 10^6^ |
| 50ppm | 5.83 x 10^6^ | 4.88 x 10^6^ | 2.65 x 10^6^ | 2.62 x 10^6^ | 2.63 x 10^6^ | 2.49 x 10^6^ | 2.44 x 10^6^ | 2.43 x 10^6^ | 2.70 x 10^6^ | 2.85 x 10^6^ |
| 70ppm | 5.83 x 10^6^ | 4.88 x 10^6^ | 1.50 x 10^6^ | 1.54 x 10^6^ | 1.27 x 10^6^ | 1.37 x 10^6^ | 1.25 x 10^6^ | 1.21 x 10^6^ | 1.29 x 10^6^ | 1.79 x 10^6^ |
| 100ppm | 5.83 x 10^6^ | 4.88 x 10^6^ | 0.45 x 10^6^ | 0.37 x 10^6^ | 0.46 x 10^6^ | 0.34 x 10^6^ | 0.41 x 10^6^ | 0.37 x 10^6^ | 0.30 x 10^6^ | 0.73 x 10^6^ |

**Table S2a**: Growth reduction of *E.coli* by the metal complexes, measured by CFU count


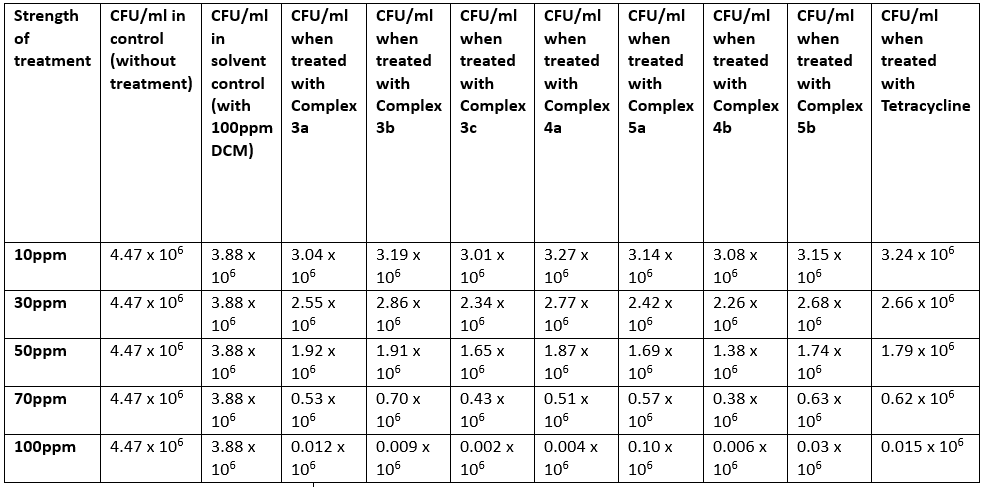


**Table S2b:** Growth reduction of *S.maltophilia* by the metal complexes, measured by CFU count


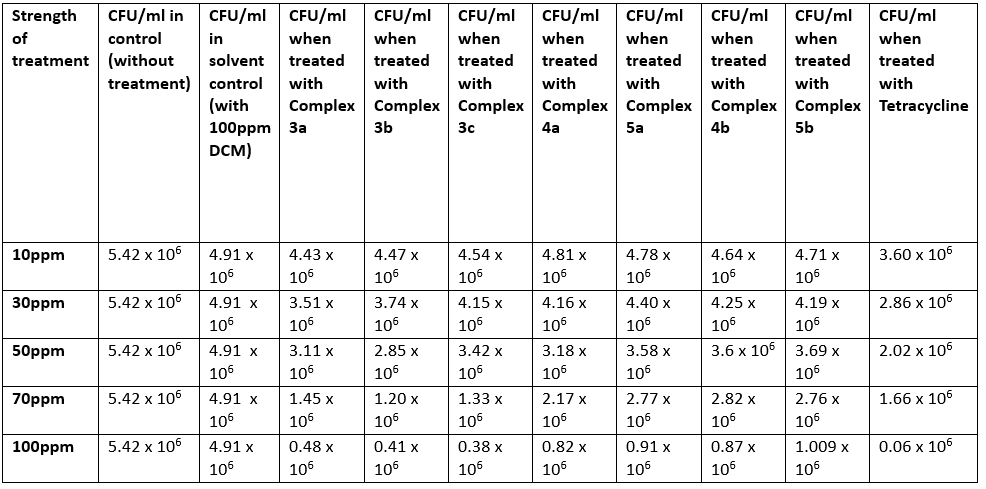


**Table S2c:** Growth reduction of *M.luteus* by the metal complexes, measured by CFU count


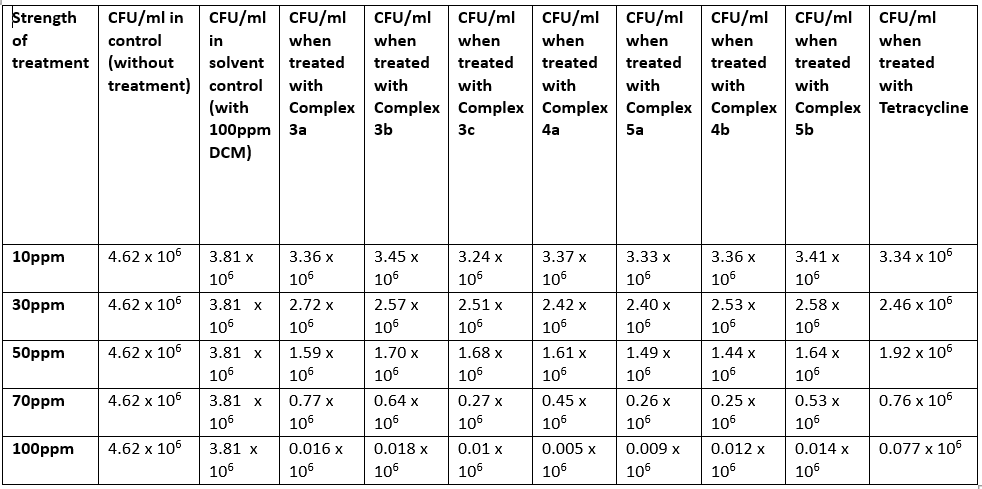


**Table S2d:** Growth reduction of *B.anthracis* by the metal complexes, measured by CFU count

**Figure S6:** Growth inhibition of *M.luteus* by ligand 1b compared to its Fe complex is nominal in compared to the respective metal complex.

**Figure S7a:** Microbial growth inhibition by Fe(II) perchlorate salt compared to respective complexes

**Figure S7b:** Microbial growth inhibition by Mn(II) salt compared to respective complexes


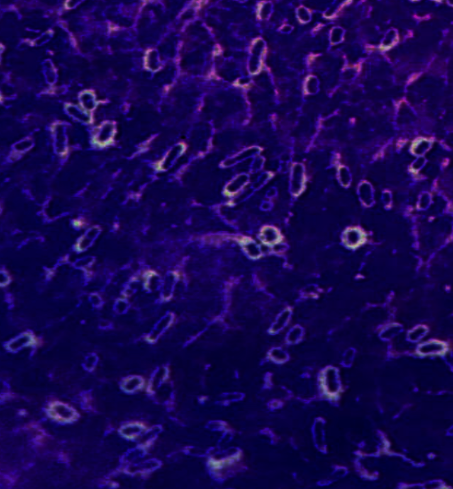


**Figure S8:** Capsule staining of *B.anthracis*. The halo zone surrounding the cells represents capsule.


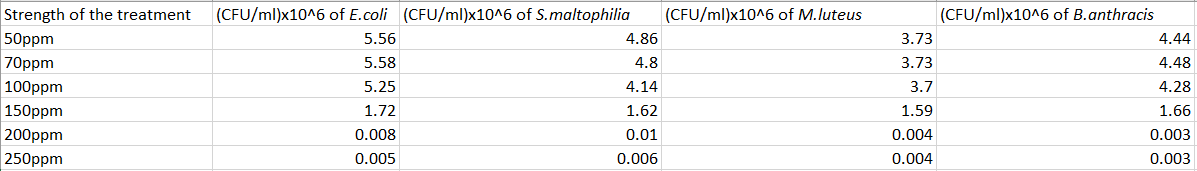


**S3a**


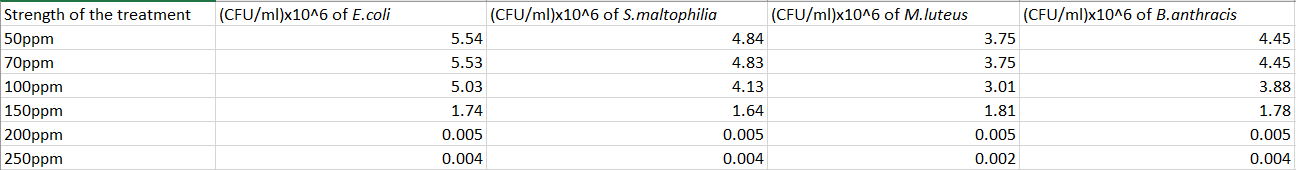


**S3b**


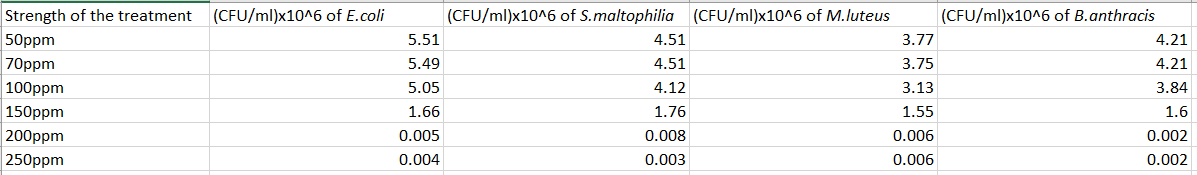


**S3c**


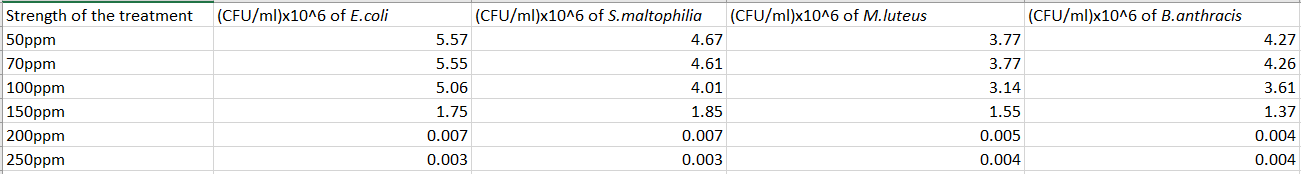


**S3d**


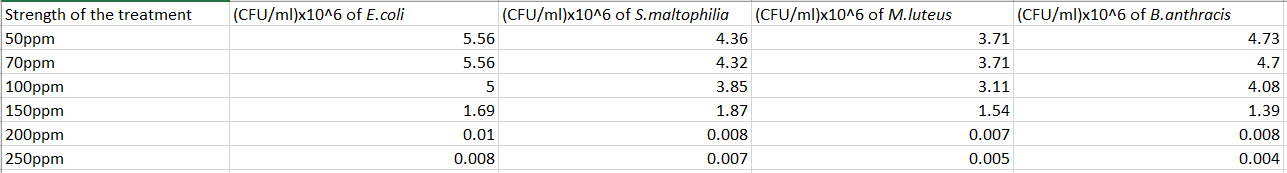


**S3e**


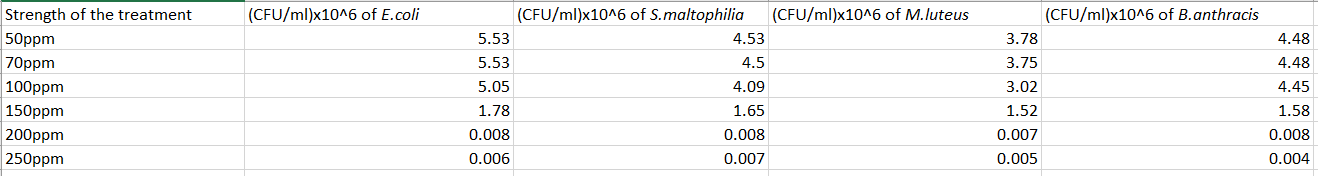


**S3f**


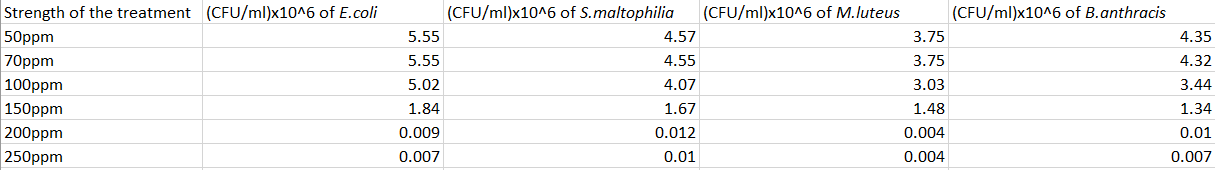


**S3g**

**Table S3:** Observation of the bactericidal effect all the seven metal complexes (table S3a, S3b, S3c, S3d, S3e, S3f, S3g represents the results of complex 3a, 3b, 3c, 4a, 4b, 5a, 5b respectively) on four bacterial strains by using CFU counts.

**Figure S9:** MDA standard curve

**Figure S10:** Formazan standard curve

| **Name of the complex** | ***E.coli*** | ***M.luteus*** | ***S.maltophilia*** | ***B.anthracis*** |
| --- | --- | --- | --- | --- |
| Peroxide conc in control | 0.0852 | 0.142 | 0.128 | 0.134 |
| Peroxide conc produced by H_2_O_2_ | 0.829 | 0.834 | 0.841 | 0.833 |
| Peroxides produced by complex 3a (mmole/L) | 0.648 | 0.637 | 0.582 | 0.614 |
| Peroxides produced by complex 3b (mmole/L) | 0.628 | 0.592 | 0.614 | 0.640 |
| Peroxides produced by complex 3c (mmole/L) | 0.529 | 0.537 | 0.544 | 0.538 |
| Peroxides produced by complex 4a (mmole/L) | 0.452 | 0.444 | 0.436 | 0.471 |
| Peroxides produced by complex 5a (mmole/L) | 0.418 | 0.427 | 0.432 | 0.425 |
| Peroxides produced by complex 4b (mmole/L) | 0.510 | 0.505 | 0.511 | 0.516 |
| Peroxides produced by complex 5b (mmole/L) | 0.429 | 0.478 | 0.488 | 0.494 |

**Table S4:** Amount (mmole/L) of ROS produced by different metal complexes in different bacterial strains.

| **Name of complex** | **Total protein concentration (microgram/ml) in *E.coli*** | **Total protein concentration (microgram/ml) in *S.maltophilia*** | **Total protein concentration (microgram/ml) in *M.luteus*** | **Total protein concentration (microgram/ml) in *B.anthracis*** |
| --- | --- | --- | --- | --- |
| **Control (without any treatment)** | 279 | *211.3* | *193.7* | *206.2* |
| [Fe^III^(Fe^II^(Ph-L^Ph^)_3_)_2_]ClO_4_ **3a^+^ClO_4_^‒^** | 82.3 | *86.8* | *73.8* | *77.4* |
| [Fe^III^(Fe^II^(Py-L^Ph^)_3_)_2_]ClO_4_ **3b^+^ClO_4_^‒^** | 78.2 | *76.7* | *86.2* | *89* |
| [Fe^III^(Fe^II^(Np-L^Ph^)_3_)_2_]ClO_4_ **3c^+^ClO_4_^‒^** | *85.4* | *82.3* | *83.4.* | *92.5* |
| [Fe^II^(Ph-L^py^)_2_] **4a** | *103* | *111* | *107* | *98.5* |
| Mn^II^(Ph-L^py^)_2_] **5a** | *95.3* | *105.6* | *114* | *116.1* |
| [Fe^II^(pTol-L^py^)_2_] **4b** | *110.4* | *98.7* | *105.8* | *103.8* |
| [Mn^II^(pTol-L^py^)_2_] **5b** | *96.1* | *127* | *120.1* | *117.6* |

**Table S5:** Total protein concentrations of all the four bacterial strains with and without the treatment with matal complexes


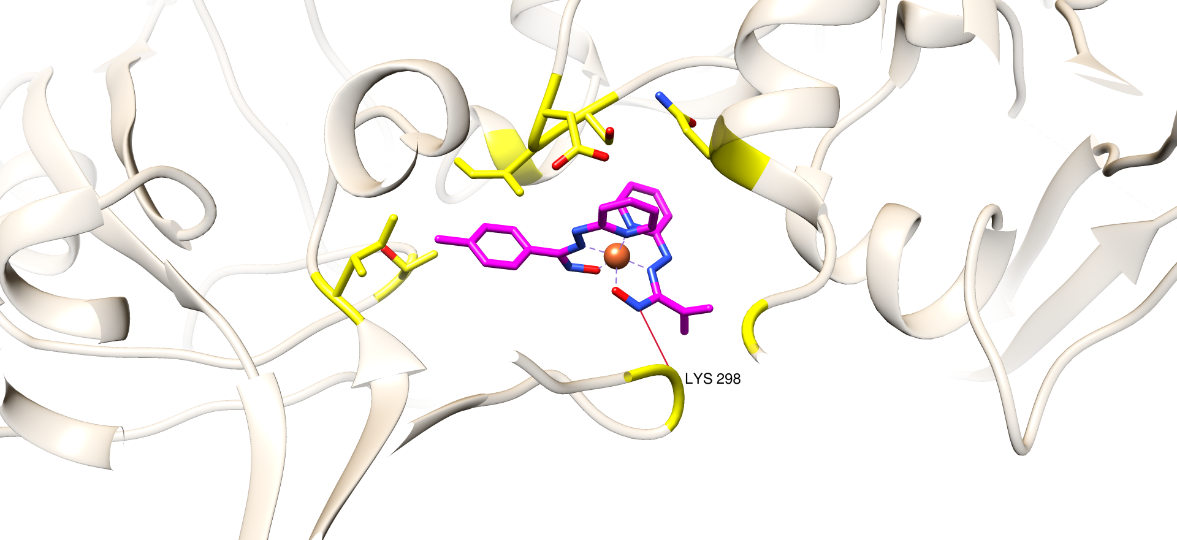


**Figure S11a:** Binding interactions of complex 5a (highlighted in magenta) and chain A of DNA Gyrase (PDB: 6RKS) subunit of *Escherichia coli* forming H bonds (shown in red) with LYS 298 (H bond restraint 0.4 Å and 120$^{\circ}$)


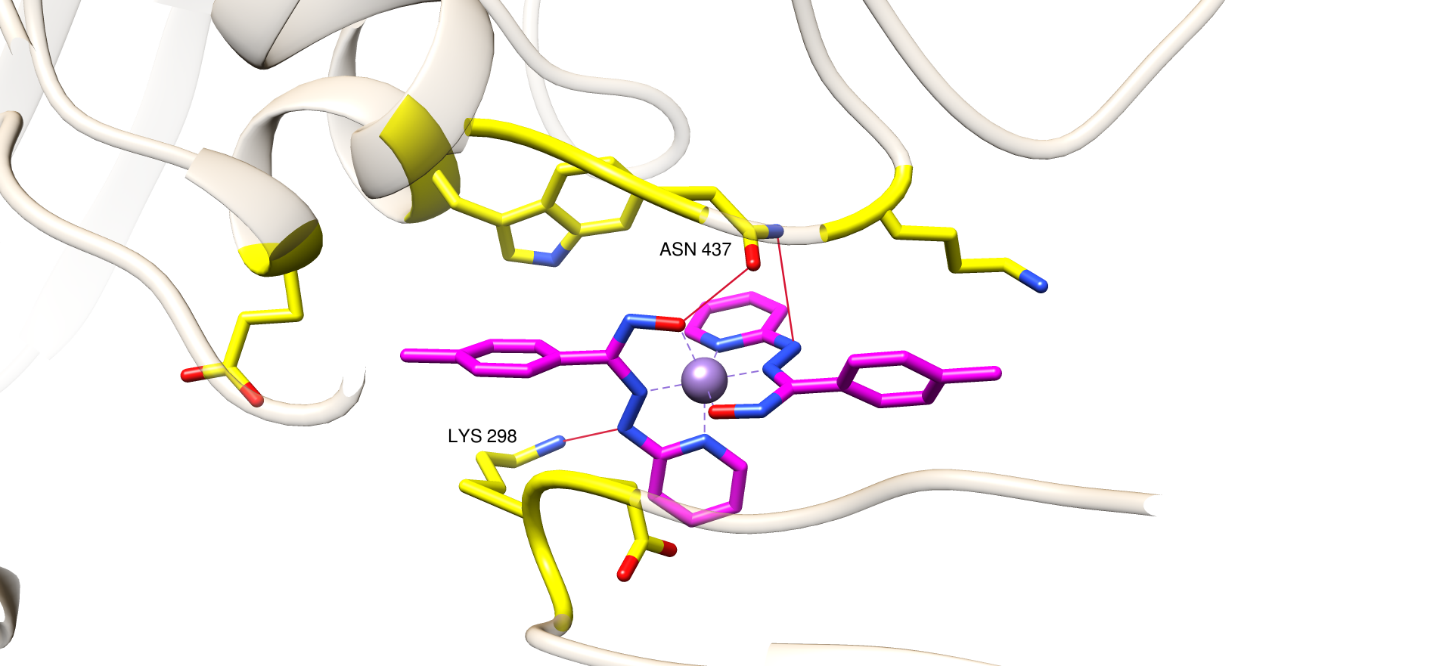


**Figure S11b:** Binding interactions of complex 5b (highlighted in magenta) and chain B and chain C of DNA Gyrase (PDB: 6RKS) subunit of *Escherichia coli* forming H bonds (shown in red) with ASN 437 and LYS 298 respectively (H bond restraint 0.4 Å and 120$^{\circ}$)


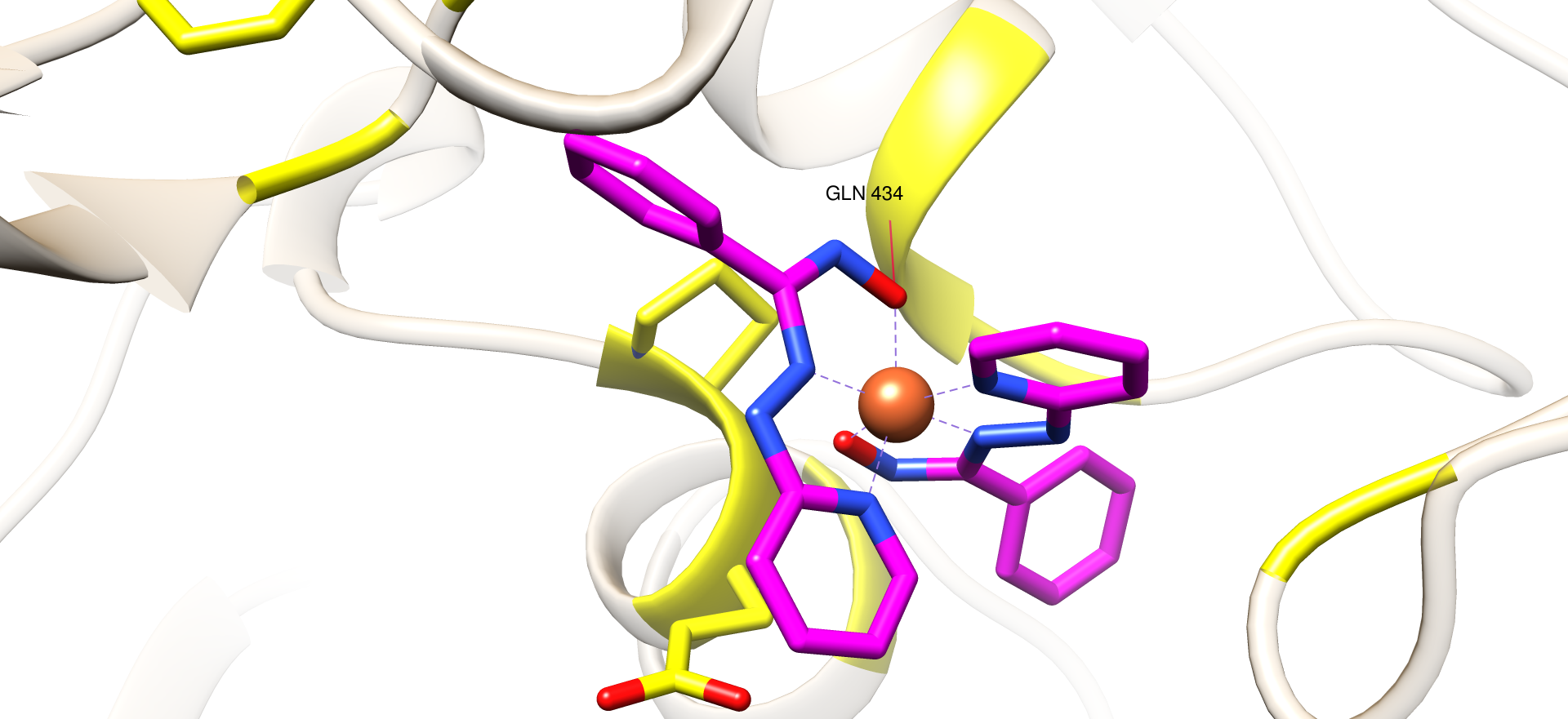


**Figure S11c:** Binding interactions of complex 4a (highlighted in magenta) and Chain D of DNA Gyrase (PDB: 6RKS) of *Escherichia coli* forming H bonds (shown in red) with GLN 434 (H bond restraint 0.4 Å and 20$^{\circ}$)


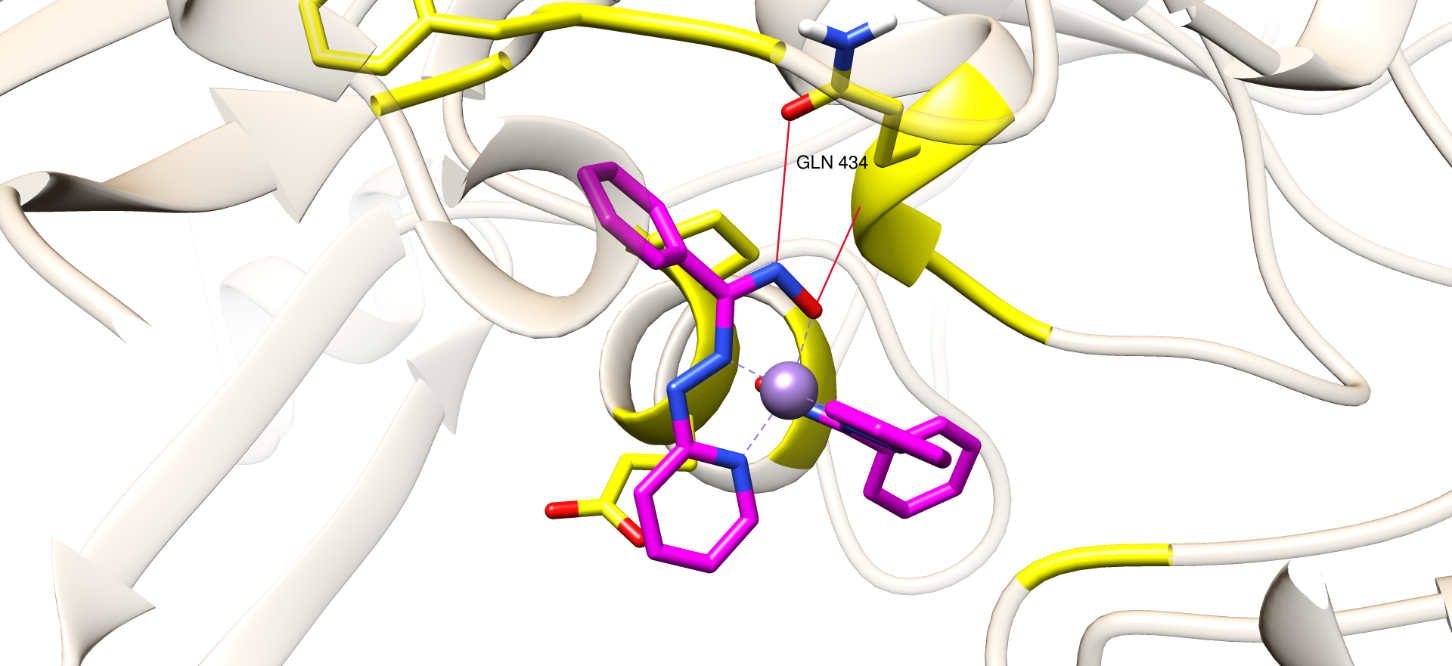


**Figure S11d:** Binding interactions of complex 4b (highlighted in magenta) and Chain D of DNA Gyrase (PDB: 6RKS) of *Escherichia coli* forming H bonds (shown in red) with GLN 434 (H bond restraint 0.4 Å and 20$^{\circ}$)

**Statistical comparison among IC50 values of different bacterial strains, using p values**

The statistical comparison among IC50 values of different bacterial strains starts with the verification, whether the data follows normal distribution ( Table S6, ESI). For that we used Shapiro Wilks test. According to the results, we rejected H_0_ hypothesis if and only if the p value (level of significance) is less than 0.05. In this experiment according to the p values *S.maltophilia* doesn’t follow normality at 5% level of significance. Hence to verify whether there is a significant difference of the average effect of the complexes, we performed Kruskal Wallis test.


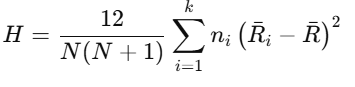


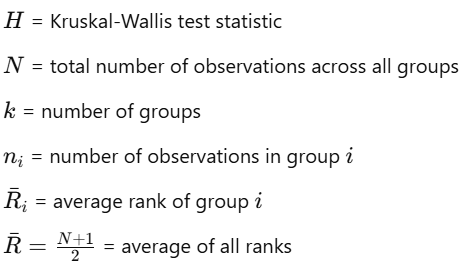


The result of the afore stated experiment (p value= 0.0305 < 0.05) suggests that there is a significant difference in IC50 values among at least one pair of bacterial strains. We used pairwise comparison to find which pair differs.

We observed that the p value for the pair *E.coli* and *S.maltophilia* is 0.0056 < 0.05 where as the p value of the rest are greater than 0.05.

Thus only *E.coli* and *S.maltophilia* shows statistically significant difference.

| **Name of data set** | **p Values** | **Interpretation** |
| --- | --- | --- |
| *E.coli* | 0.379 > 0.05 | Normally distributed |
| *M.luteus* | 0.178 > 0.05 | Normally distributed |
| *S.maltophilia* | 0.023 < 0.05 | Not Normally distributed |
| *B.anthracis* | 0.175 > 0.05 | Normally distributed |

**Table S6:** Normality test of IC50 values of different bacterial strains by the metal complexes using Shapiro Wilks method.
